# Expanding the understanding of majority-bias in children’s social learning

**DOI:** 10.1038/s41598-022-10576-3

**Published:** 2022-04-25

**Authors:** Anne Sibilsky, Heidi Colleran, Richard McElreath, Daniel B. M. Haun

**Affiliations:** 1 Department of Comparative Cultural Psychology, Max Planck Institute for Evolutionary Anthropology, Leipzig, Germany; 2 Leipzig Research Center for Early Child Development, Leipzig University, Leipzig, Germany; 3 BirthRites Independent Max Planck Research Group, Max Planck Institute for Evolutionary Anthropology, Leipzig, Germany; 4 Department of Human Behavior, Ecology and Culture, Max Planck Institute for Evolutionary Anthropology, Leipzig, Germany

**Keywords:** Cultural evolution, Human behaviour

## Abstract

Prior experiments with children across seven different societies have indicated U-shaped age patterns in the likelihood of copying majority demonstrations. It is unclear which learning strategies underlie the observed responses that create these patterns. Here we broaden the understanding of children’s learning strategies by: (1) exploring social learning patterns among 6–13-year-olds (*n* = 270) from ethnolinguistically varied communities in Vanuatu; (2) comparing these data with those reported from other societies (*n* = 629), and (3) re-analysing our and previous data based on a theoretically plausible set of underlying strategies using Bayesian methods. We find higher rates of social learning in children from Vanuatu, a country with high linguistic and cultural diversity. Furthermore, our results provide statistical evidence for modest U-shaped age patterns for a more clearly delineated majority learning strategy across the current and previously investigated societies, suggesting that the developmental mechanisms structuring majority bias are cross-culturally highly recurrent and hence a fundamental feature of early human social learning.

## Introduction

Across societies, children acquire a wide range of competencies as they adapt to their physical, social and cultural environments^1,2^ through individual and social learning. Social learning “…refers to any time an individual’s learning is influenced by others,… “, and is frequently contrasted to individual learning, in which “individuals learn by observing or interacting directly with their environment”^3^^(p12)^. Social learning avoids the costs of learning by trial and error^4,5^ and hence plays a key role in cognitive and behavioral development^6^. There are many different modes of social learning, each generating different patterns of cultural variation and change.

One mode of learning in particular is a majority-bias, here defined as the tendency of an individual to preferentially adopt behaviors demonstrated by a majority^7^. In the context of cultural evolution, a strong form of majority-biased learning, in which the probability of adopting the majority behavior is higher than the proportion of the majority demonstrators who perform it (so-called “conformist transmission”), is argued to be related to cultural diversity^8^. While cultural diversity does not require majority-biased learning, disproportionately high copying of the majority minimizes variation within and maximizes variation between groups of individuals ensuring long term stability of population-level variation. Hence variable levels of cultural diversity across societies might be related to population level variation in individuals’ majority bias. We aim to test this hypothesis by comparing children’s social learning in a society with extensive linguistic and cultural variation to that of children in previously studied societies.

Previous studies have reported that, across cultures, children copy the majority when confronted with opposing behaviors or opinions of a majority and a minority of individuals ^7,9–16^. Children either interact with an unknown apparatus to obtain a reward after seeing a majority and a minority of models interacting with it ^7,11–14^ or they are asked to tell the name or function of an unknown object after they heard descriptions from them^9,10,17^. Here, we apply the former design and neglect the latter as language depends on a consensus of the majority to be functional^18^, and constitutes a special case of social learning.

When children use a device in the same way that a majority of children have used it previously, these observed responses could be driven by several underlying strategies beyond majority-bias. It is therefore unclear which learning mechanisms generate the patterns previously found. For example, a tendency to copy a random demonstrator would amount to the same outcome as a majority bias, since it is consistently afforded by the majority^19^. One study investigating strategies by systematically manipulating the degree of informant consensus showed a shift from anti-conformist to conformist tendencies in non-naïve children (i.e., children could know the correct answer through asocial information) between 3 and 7 years^16^ indicating developmental shifts in children’s strategy use. Here, we are interested in the development of majority bias of *naïve* children and differentiate between observable responses on the one hand and underlying social learning strategies on the other.

**Figure 1:**
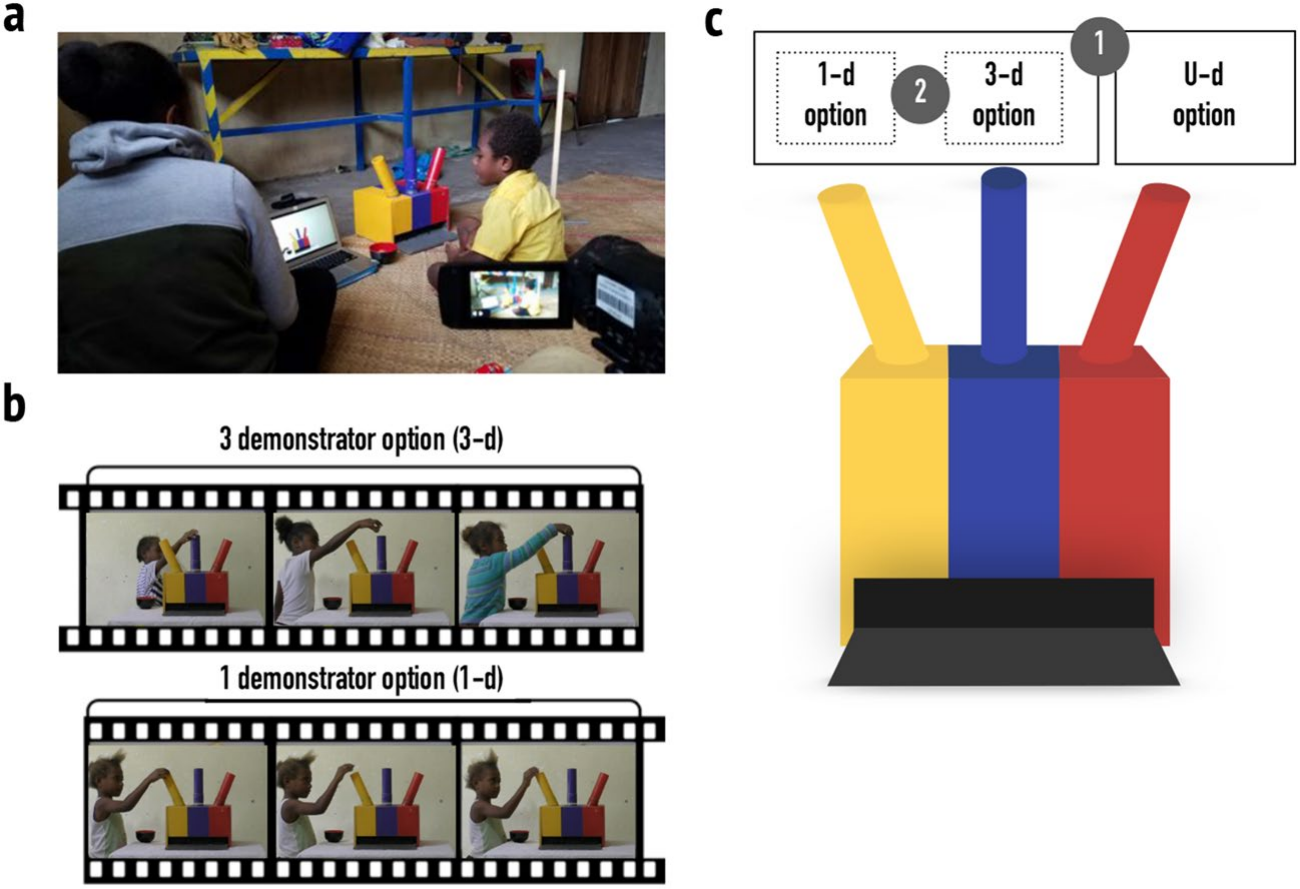
Study Design and Setup. Panel (**a**) shows the study setup (photo by Jule Wolf), panel (**b**) shows an example demonstration video, and panel (**c**) shows the possible response options (1-d option, 3-d option, U-d option), in this counterbalancing version in the colors yellow, blue and red, respectively. On the level of observed responses, we describe (1) children’s tendency to respond following the demonstrated options by comparing the proportion of children who chose one of the two demonstrated options with those who chose the undemonstrated option (solid lines), and (2) children’s “3-demonstrator” responses by comparing the proportion of children who chose the 3-d option with those who chose the 1-d option (dotted lines).

In the most comprehensive study to date on social learning in naïve children, van Leeuwen and colleagues^13^ investigated observed responses among 4 to 14 year olds from seven societies: Central African Republic, Brazil, Germany, Namibia, Indonesia, Kenya, and Zambia. Children faced a simple decision-making task (see also^7^): they were asked to approach a box with three different colored subsections (Fig. 1a) and to place a ball into a specific subsection, yielding a reward. Before making their decision, children watched a video (Fig. 1b) in which three children demonstrated the same option once each (3 demonstrator option: 3-d option) and one child that demonstrated another option three times (1 demonstrator option: 1-d option). The order of the 3-d and 1-d demonstrations was counterbalanced. The third subsection of the box was never demonstrated (undemonstrated option: U-d option). After watching the video, the focal child responded, either following one of the two demonstrated options (3-d or 1-d), or the undemonstrated option (U-d; Fig. 1c).

Children’s observed responses across societies on average mostly followed one of the two demonstrated options (3-d or 1-d), with few following the U-d option. The proportions of observed responses following one of the two demonstrated options relative to the U-d option varied by society. In three societies they increased with age, in the other four they decreased. In contrast, the proportions of observed responses following the 3-d relative to the 1-d option did not differ significantly across societies. The age-related pattern of observed responses following the 3-d relative to the 1-d option recurred across societies: relatively younger and older children mostly responded following the 3-d option, whereas children at intermediate ages tended to follow the 1-d option more often. Based on these findings, the researchers suggested that children’s tendency to copy the majority follows a U-shaped developmental trajectory across societies^13^.

We argue that two major limitations constrain inferences about the ontogeny of social learning from this research. First, small sample sizes in several societies and a frequentist statistical approach that is sensitive to this problem limit the robustness of these results. Second, van Leeuwen and colleagues^13^ took children’s observed responses to be direct evidence of a majority or minority bias (strategy), when in fact multiple strategies are consistent with the observed responses. While they experimentally controlled for aspects of underlying strategies (frequency bias, order bias, gender bias), some alternative strategies are impossible to distinguish based on observed responses alone. For example, an individual observed response that appears guided by the 3-d option in the task could be equally based on copying the majority of demonstrators (majority bias) or a random demonstrator (randomly copying one of four demonstrators). Similarly, several strategies could also underlie observed responses following the 1-d and U-d options.

Here, we address these limitations by examining observed responses in the same scenario among a sample of children living in a highly linguistically and culturally diverse context, the Pacific island-nation of Vanuatu. Using the same paradigm as van Leeuwen and colleagues^13^ (see Fig. 1), we collected data on a large number of children (*n* = 270) from five distinct communities within one island. We furthermore assessed the likelihood that a range of different strategies account for children’s observed responses in both Vanuatu and the cross-cultural sample from van Leeuwen and colleagues^13^.

Investigating social learning among ni-Vanuatu children is particularly valuable because of the high indigenous linguistic and cultural diversity, and the fact that these differences are salient markers of between and within community identity. Besides the three official languages: Bislama, French, and English, 138 indigenous languages are spoken, all part of the Oceanic sub-group of the Austronesian language family^20^. The population numbers approximately 300,000 inhabitants^21^. Assuming language groups can approximate the number of cultural groups^22^, Vanuatu’s linguistic diversity is indicative of extensive cultural diversity within a small geographic area. Moreover, ethnopsychological research indicates that social values such as community, cooperation and an orientation towards others are important^23^ and that obedience, norm compliance, and respect for elders are emphasized^24^. In line with these norms, experimental research indicates that ni-Vanuatu adults are more likely than US adults to endorse highly imitating children as “well-behaved”^25^ (but see ^26^ for high endorsement by children and adolescents from both societies). ni-Vanuatu children, in turn, have been shown to imitate with higher fidelity than US children in an instrumental task^27^.

Given the high level of cultural diversity, the value of orienting oneself towards others, and the empirical evidence of high levels of imitation, we expected ni-Vanuatu children to respond to social learning tasks by rarely following the U-d option and to disproportionately frequently follow the 3d-option in relation to other, previously tested societies. Our first goal is to describe the observed responses of ni-Vanuatu children in relation to the prior cross-cultural sample by applying the original coding of van Leeuwen and colleagues^13^ for maximal comparability. Our second goal is to refine previous results by modeling a plausible range of strategies underlying observed responses from children in Vanuatu and previous samples^13^.

## Results

We present the results in three parts. First, we describe the pattern of observed responses in the ni-Vanuatu sample without explaining them. Second, we compare the pattern of responses in the ni-Vanuatu sample to previous cross-cultural samples. Finally, we analyze underlying learning strategies in the full sample of ni-Vanuatu and previous cross-cultural data. All data and analysis scripts are available on the Open Science Framework (https://osf.io/qcs7n).

### Description of the ni-Vanuatu sample

**Figure 2:**
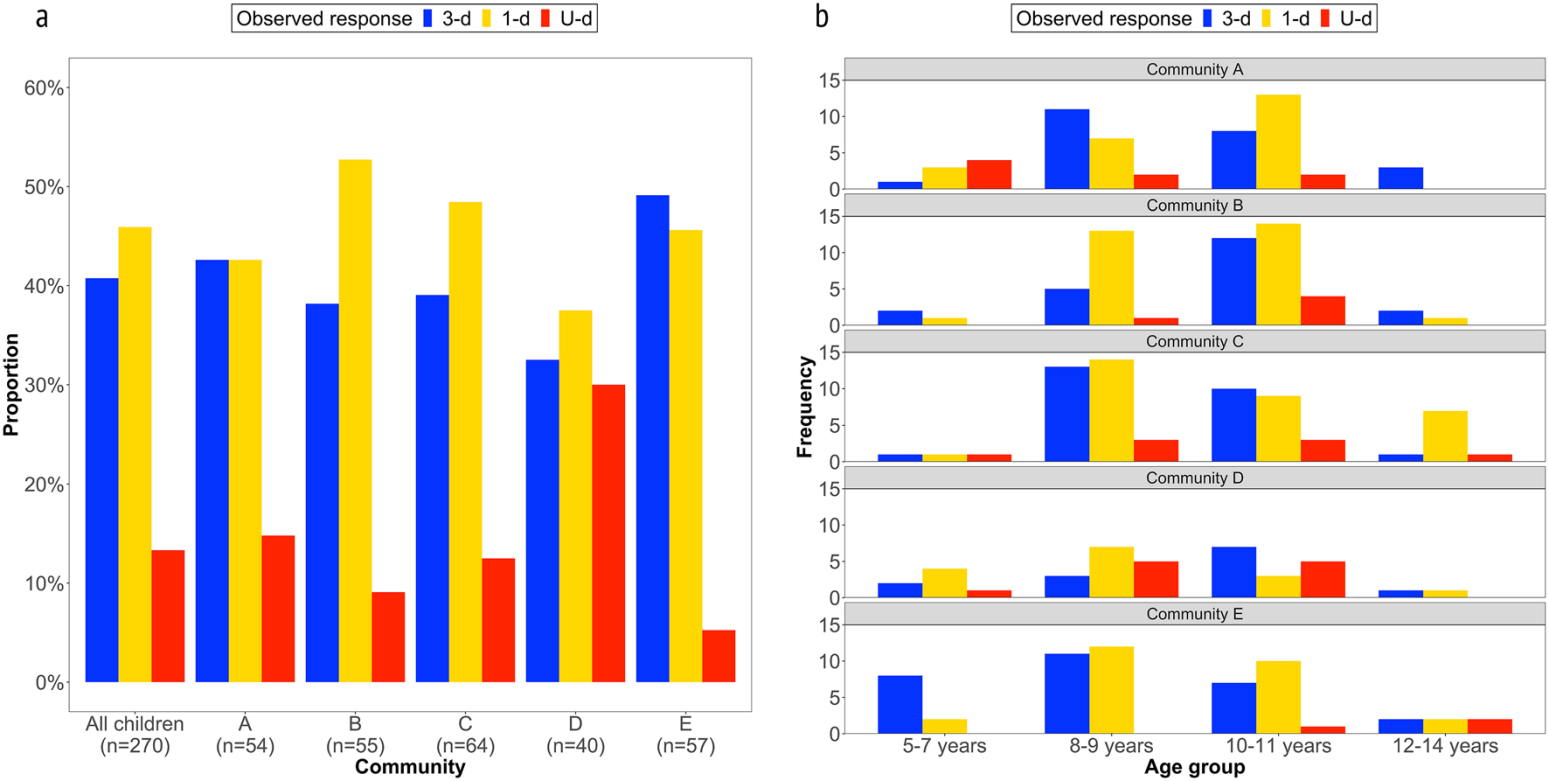
Descriptive distribution of observed responses. Children chose a demonstrated option more frequently than an undemonstrated option across communities, with little variation by age across communities. Panel (**a**) shows response distributions following the 3-d option (blue), 1-d option (yellow) and U-d option (red) across the five communities. Panel (**b**) shows the frequencies of each observed response by community and age group.

41% of ni-Vanuatu children’s observed responses followed the 3-d option, 46% the 1-d option and 13% the U-d option (Fig. 2a, see Supplementary Results 1.1 for the frequencies). This pattern is consistent within all communities in our Vanuatu sample except for one (community D), in which the three options have a similar frequency (3-d option: 32%, 1-d option: 38%, U-d option: 30%). Most often, one of the two demonstrated options was chosen by ni-Vanuatu children. There was little variation between communities in the distributions of observed responses by age groups (Fig. 2b). Exceptions are community E, in which many young children seem to follow the 3-d option, and community C, in which many old children seem to follow the 1-d option. However, due to the small sample sizes in these age groups, caution should be exercised with this result. Averaged across age, children did not follow the 3-d option more frequently, but rather, and consistent with previous research, their observed responses tend to follow the first demonstration: the odds of following an option were 9.4 times larger when it was demonstrated first.

We examined whether children’s understanding of the demonstration video (i.e., their performance on a manipulation check), was associated with their observed responses. 221 children (82% of all 270 children) correctly remembered which colours were used by the demonstrators. Of these, 107 children (40% of all 270 children) additionally correctly recalled that the 3-d demonstrators were more children than the 1-d demonstrator. We found no associations between children’s understanding of the demonstration video and their observable responses (see Supplementary Results 1.2).

### Comparison of the ni-Vanuatu sample with previously published data

To compare ni-Vanuatu children’s observed responses with existing data from eight other societies^13^, we used a Bayesian multilevel model with varying intercepts and age trends across societies. See the precise model definition in Methods. Note that in the original analysis by van Leeuwen and colleagues^13^, Samoan children were excluded because they were judged to be highly influential during leave-one-out cross-validation (see Supplemental note 2 from ^13^). We included the Samoa sample in our analysis.

**Figure 3:**
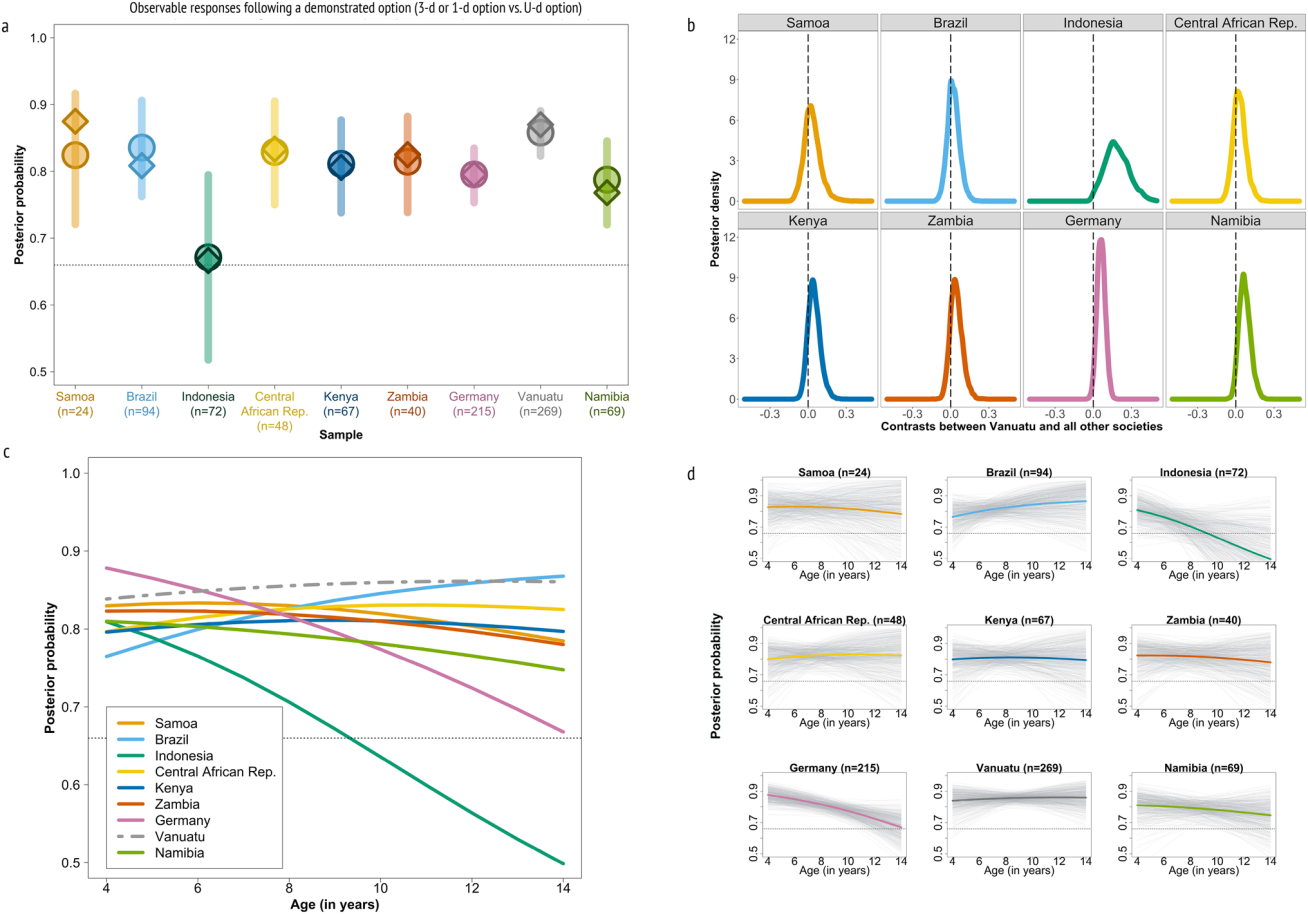
Predictions of the Bayesian multilevel models for observed responses following one of the demonstrated options vs. the undemonstrated option in Vanuatu and eight other societies. Panel (**a**) shows the raw data overlaid on the probability of selecting one of the two demonstrated options vs. the undemonstrated option based on multilevel model estimates. Circles and vertical lines represent the multilevel model estimates and 89% confidence intervals, rhombuses represent the raw proportions in the data. The horizontal dotted line represents the expected proportion if children’s observed responses were random (probability of 0.66). The probability of observed responses following a demonstrated option is consistently greater among ni-Vanuatu children compared to children from the other societies. Panel (**b**) shows posterior density distributions for each society where each is a deviation from the Vanuatu data. This deviation is mostly positive for the probability of observed responses following a demonstrated option. Panel (**c**) shows society-specific age-patterns of children’s observed responses following one of the demonstrated options. ni-Vanuatu children’s observed responses show a constant level of following demonstrated options by age. The horizontal dotted line represents the expected proportion if children’s observed responses were random (probability of 0.66). Panel (**d**) shows society-specific age-trajectories of children’s reliance on a demonstrated option vs. the undemonstrated option. Horizontal dotted lines represent the expected proportion if children’s observed responses were random (probability of 0.66). The light gray lines are 500 lines sampled from the posterior distribution showing the uncertainty of the predicted trajectories.

Figure 3a shows the overall probability of selecting one of the demonstrated options (3-d or 1-d) vs. the U-d option in each society based on this model (see Supplementary Results 2.1 for the response distribution and 2.2 for model coefficients). Averaged over age, ni-Vanuatu children’s observable responses followed a demonstrated option more often than children from eight other societies. To calculate the expected difference between the ni-Vanuatu distribution and those of the other eight societies, we calculated contrasts and their respective 89%-highest posterior density interval (HPDI). These indicate (Fig. 3b) that the probability of observing a response following a demonstrated option is higher among ni-Vanuatu children compared to children from Indonesia (*HPDI* = 0.03–0.33), Germany (*HPDI* = 0.01–0.11) and Namibia (*HPDI* = 0.00–0.14). Deviations from other societies are in the same direction, but with little evidence the differences are robust: Samoa (*HPDI* = − 0.07–0.13), Brazil (*HPDI* = − 0.06–0.09), Central African Republic (*HPDI* = − 0.05–0.11), Kenya (*HPDI* = − 0.03–0.12), and Zambia (*HPDI* = − 0.03–0.12).

The tendency for observed responses to follow either of the two demonstrated options appears to be fairly constant across age groups among ni-Vanuatu children (Fig. 3c and d). This contrasts with declining trends over age in Germany and Indonesia, a broad trend towards declining probabilities in most of the other societies, and moderately increasing trends in Brazil. Observed responses following demonstrated options tend to be more likely over most of the age-range in Vanuatu compared to all other societies, though the evidence for a difference is rather weak. Only among over-8-year-old children is there robust evidence that ni-Vanuatu children are more likely to choose a demonstrated option than German or Indonesian children (see Supplementary Results 2.5 for age-dependent contrasts).

**Figure 4:**
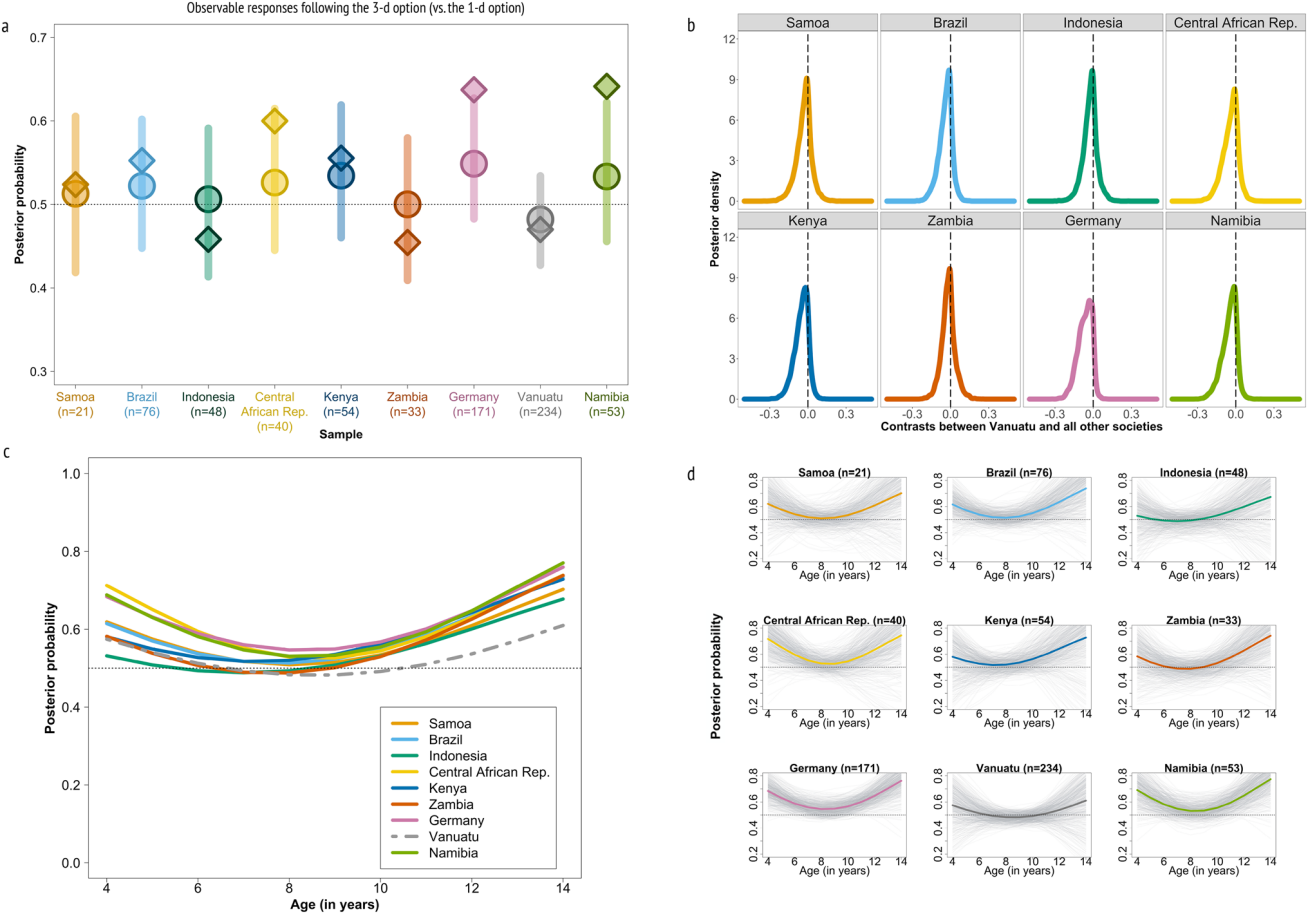
Predictions of the Bayesian multilevel models for observed responses following the 3-d option vs. the 1-d option in Vanuatu and eight other societies. Panel (**a**) shows the raw data overlaid on the probability of following the 3-d option vs. the 1-d option based on multilevel model estimates. Circles and vertical lines represent the multilevel model estimates and 89% confidence intervals, rhombuses represent the raw proportions in the data. The horizontal dotted line represents the expected proportion if children’s observed responses were random (probability of 0.5). The probability of following the 3-d option vs. the 1-d option option is consistently lower among ni-Vanuatu children compared to children from the other societies. Panel (**b**) shows posterior density distributions for each society where each is a deviation from the Vanuatu data. This deviation is mostly negative for the probability of observed responses following the 3-d option. Panel (**c**) shows society-specific age-patterns of children’s observed responses following the 3-d option. ni-Vanuatu children’s observed responses show a tendency towards a U-shaped age-pattern of following the 3-d option. The horizontal dotted line represents the expected proportion if children’s observed responses were random (probability of 0.5). Panel (**d**) shows society-specific age-trajectories of children’s reliance on the 3-d vs. the 1-d option. Horizontal dotted lines represent the expected proportion if children’s observed responses were random (probability of 0.5). The light gray lines are 500 lines sampled from the posterior distribution showing the uncertainty of the predicted trajectories.

Figure 4a shows the overall probability of choosing the 3-d vs. the 1-d option in each society (see Supplementary Results 2.1 for the response distribution and 2.2 for the model coefficients). Here we find weak evidence that averaged over age ni-Vanuatu children are the least likely to select the 3-d vs. the 1-d option compared to children from all other societies. Though most of the probability mass of the difference between Vanuatu and all other societies is below zero, pairwise contrasts (Fig. 4b) indicate that the differences are neither large nor precise (Samoa: *HPDI* = − 0.13–0.06, Indonesia: *HPDI* = − 0.11–0.06, Brazil: *HPDI* = − 0.12–0.04, Central African Republic: *HPDI* = − 0.14–0.05, Kenya: *HPDI* = − 0.15–0.03, Zambia: *HPDI* = − 0.11–0.06, Germany: *HPDI* = − 0.15–0.01, Namibia: *HPDI* = − 0.14–0.03).

We do not have data for very young or very old ni-Vanuatu children because of our more restricted age-range (6–13 years). The model predictions show, however, that our data are consistent with a modest U-shaped age pattern of observed responses following the 3-d option (Fig. 4c and d), in which the probability starts at 57% (89% percentile interval (PI) [0.34;0.78]), then declines to 48% (89% PI [0.43;0.53]) and then steadily increases to 61% (89% PI [0.31;0.83]). Note, however, that evidence for this U-shaped pattern is absent when conducting the analysis with the ni-Vanuatu data separately, indicating that our results are driven largely by the inclusion of past data (see Supplementary Results 2.3 for a separate analysis of the ni-Vanuatu sample). Further, other shaped patterns cannot be ruled out. Varying evidence for a U-shaped age pattern is also found in the other eight societies, with stronger evidence for some societies such as Germany and weaker evidence for others such as Indonesia (see Supplementary Results 2.4 for society-specific 89% HDPIs of the quadratic age term). Age-specific contrasts show weak evidence of a difference between ni-Vanuatu children’s probabilities of choosing the 3-d option and the other samples. However, there is a general trend for ni-Vanuatu children to be less likely to choose the 3-d compared to all other societies across most of the age range (see Supplementary Results 2.5 for age-dependent contrasts).

### Learning strategies

To get a clearer picture of the extent to which children’s observed responses following the 3-d options reflect a strategy to follow the majority (majority bias) we defined theoretical probabilities for a range of possible underlying strategies and modelled the likelihood with which they account for observed responses. Table 1 describes the strategies we explored, and the probability that a child using a given strategy would select each observable response. See the precise model definition in Methods. Our results are of course conditional on this a-priori set of strategies.

**Table 1:** Expected probabilities of observed responses in the experiment, based on an assumed underlying strategy.

	Strategy	Explanation	Expected probability for each option		
			3-d	1-d	U-d
1)	Majority bias	Copy the majority behavior	1	0	0
2)	Minority bias	Copy the minority behavior	0	1	0
3)	Undemonstrated	Choose the undemonstrated option	0	0	1
4)	Primacy bias	Copy the option of the video first shown	whatever is shown first: 1, the other: 0		0
5)	Random demonstrator	Copy according to the number of demonstrators	¾	¼	0
6)	Random instance	Copy according to the number of demonstrations	½	½	0
7)	Random	Random decision or copy according to a randomly distributed criterion	1/3	1/3	1/3

We then computed the posterior distribution of this set of strategies based on the observed frequencies of the 3-d, 1-d and U-d options in our data and that of van Leeuwen and colleagues^13^. This means that in contrast to the previous two steps in which we operationalized observed responses as binary responses, we used a trinomial-outcome model with three possible outcomes: 3-d option, 1-d option and U-d option.

**Figure 5:**
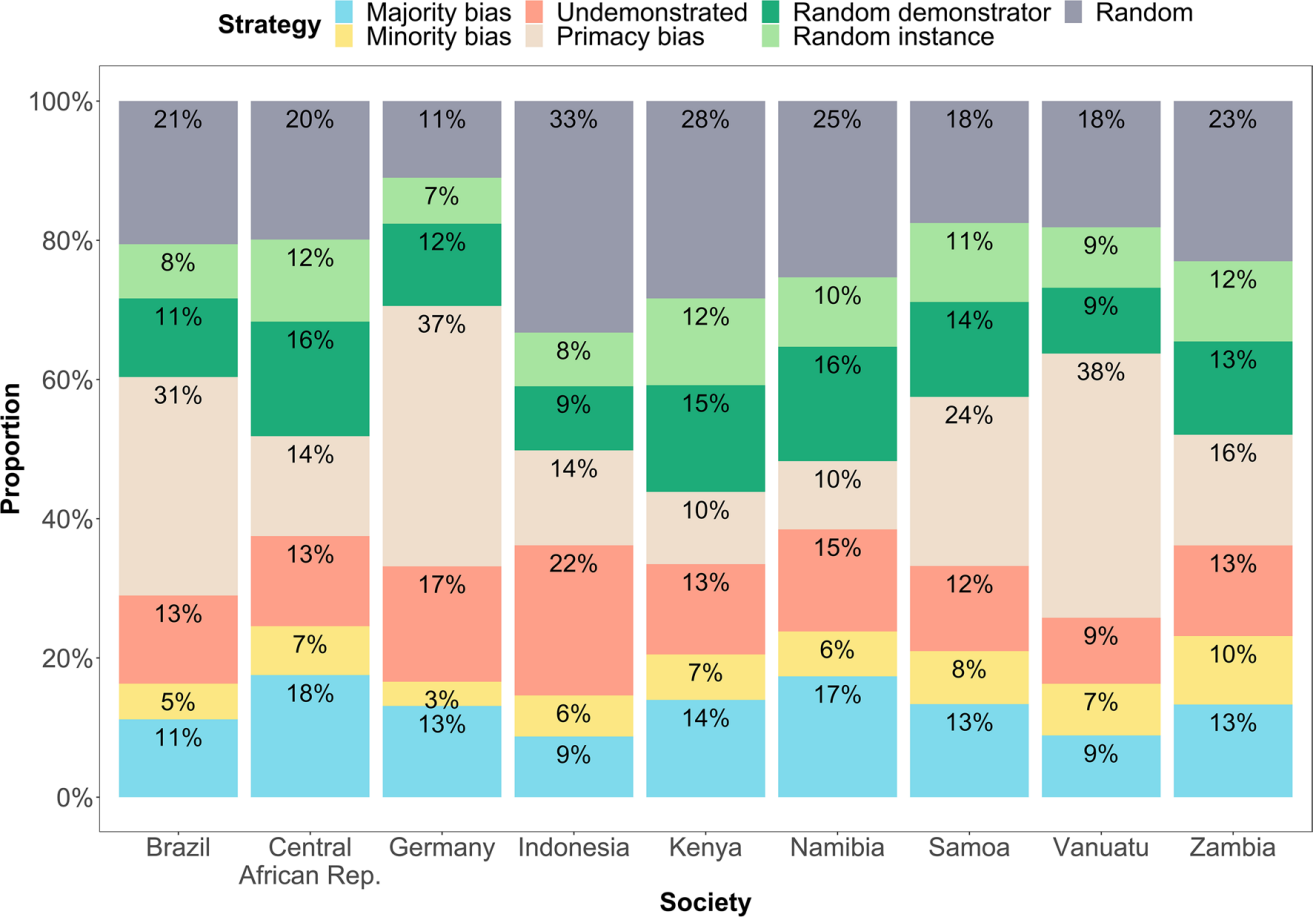
Inferred posterior mean probabilities of the seven selected learning strategies by society averaged over age.

In the following, we provide a summary of the posterior mean probabilities of each strategy and classify them in the societies studied. Note that this classification needs to be treated with caution as the boundaries of these probabilities are associated with considerable uncertainty. We find that the posterior mean probability that a child uses a majority-biased strategy varies between societies from 9% (89% PI [0.00;0.31]) to 18% (89% PI [0.00;0.57]) (Fig. 5, see Supplementary Results 3.1 for all values). Only in the Central African Republic (18%; 89% PI [0.00;0.57]) does it appear to be the most likely strategy. The posterior mean probability of a minority biased strategy varies between societies from 3% (89% PI [0.00;0.10]) to 10% (89% PI [0.00;0.31]). Across societies we infer this to be the least likely strategy. The strategy of following the undemonstrated option varies between societies from 9% (89% PI [0.01;0.20]) to 22% (89% PI [0.01;0.52]), being the most prevalent strategy in Indonesia (22%; 89% PI [0.01;0.52]).

In fact, a wider range of strategies is needed to fully explain children’s responses. We find that the probability that a child shows a primacy bias ranges from 10% (89% PI [0.01;0.25]) to 38% (89% PI [0.12;0.57]) and is in fact the most likely strategy in Brazil (31%, 89% PI [0.08;0.53]), Germany (37%, 89% PI [0.13;0.60]), Samoa (24%, 89% PI [0.03;0.51]) and Vanuatu (38%, 89% PI [0.12;0.57]). The probability of using a random demonstrator strategy (the probability of copying is proportional to the number of demonstrators) varies between societies from 9% (89% PI [0.00;0.37]) to 16% (89% PI [0.00;0.61]). It is the most prevalent strategy in Kenya (15%, 89% PI [0.00;0.55]) and Namibia (16%, 89% PI [0.00;0.61]). The probability that a child uses a random instance strategy (the probability of copying is proportional to the number of demonstrations) varies between societies from 7% (89% PI [0.00;0.22]) to 12% (89% PI [0.00;0.44]). Finally, the probability of random copying (where the probability of copying is truly random, i.e., proportional to the number of options shown; this category is more of an umbrella term for randomly distributed criteria such as favorite color than a strategy) varies between 11 (89% PI [0.00;0.38]) and 33% (89% PI [0.01;0.87]) across societies.

**Figure 6:**
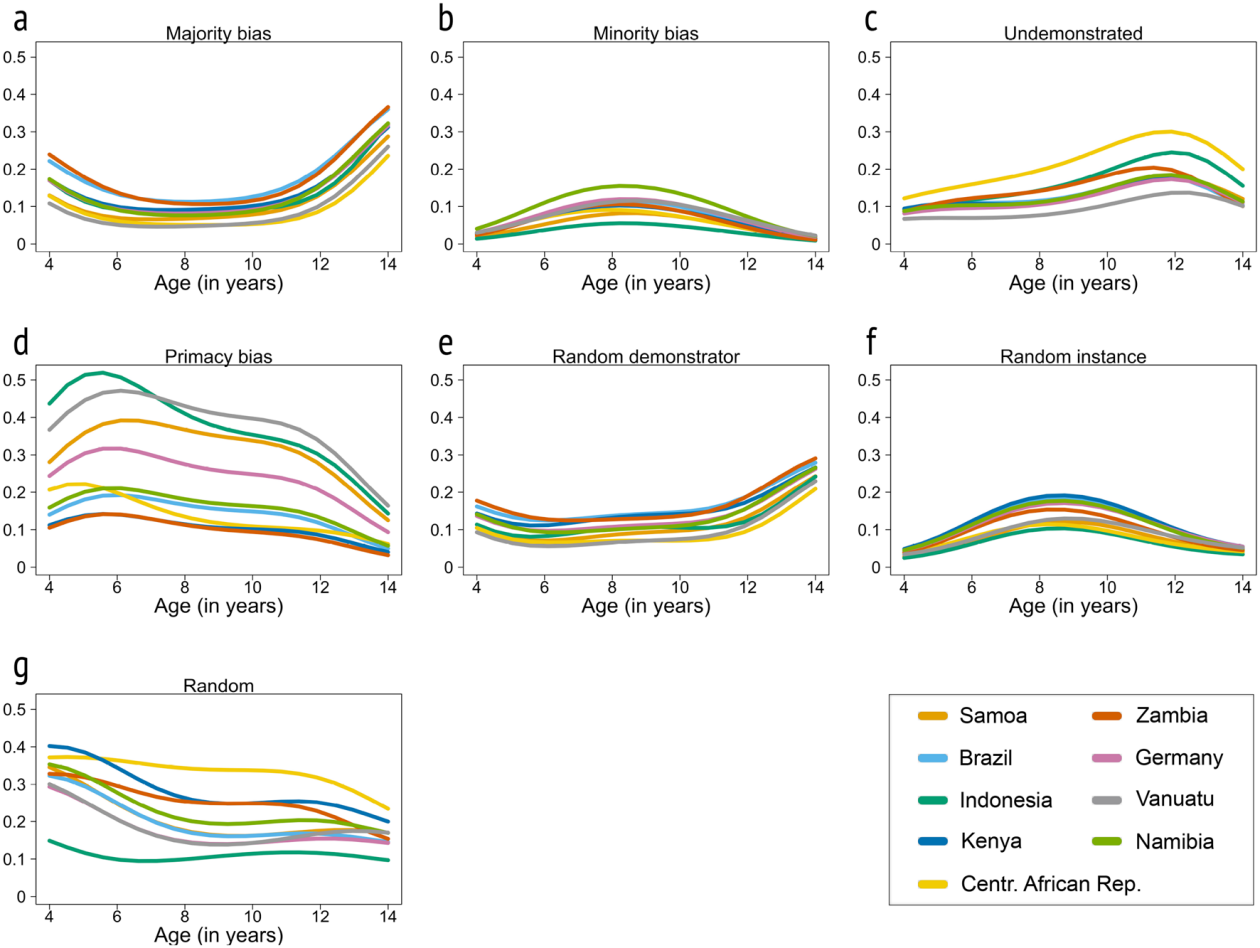
Age patterns of the seven inferred learning strategies by society and across age based on posterior distributions inferred of a Bayesian trinomial-outcome model. The majority bias (**a**) and the random demonstrator strategy (**e**) follow a U-shaped age pattern, while the minority bias (**b**) and random instance strategy (**f**) follow an inverted U-shaped age pattern. The strategy to use the undemonstrated option increases over age before it declines (**c**), the primacy bias decreases (d). Throughout the investigated ages, some data remain randomly distributed (**g**).

We also estimated the likely age patterns of the inferred strategies in the different societies (Fig. 6, see Supplementary Results 3.2 for alternative illustrations). Across societies the age-trend of the probability of the majority bias follows a modest U-shaped pattern (see Fig. 6a). A predictive model comparison using Pareto-smoothed importance sampling^28^ prefers the quadratic age model (PSIS score 1732) to a linear age model (PSIS score 1736). Although the superiority of the quadratic model is clear, the score difference remains small because most of the variation in the data is not associated with age. Together with the fact that most of the posterior probability in the quadratic model favours curvilinear relationships between strategy and age, there is no statistical reason to prefer a linear function. However, the evidence indicates that the curvilinear relationship with age could be strong or rather weak or even show another form of relationship (Supplementary Results 3.3 for the uncertainty of the U-shape).

The probability of minority bias follows an inverted U-shaped age pattern (see Fig. 6b). The probability of the strategy of choosing the undemonstrated option increases with age before declining (Fig. 6c).

Considering the probabilities of the other strategies in the model, there is again developmental variation: the chances of a primacy bias tend to decrease with age (Fig. 6d), the random demonstrator strategy appears to slightly increase with age (Fig. 6e) and the random instance strategy follows an inverted U-shape over age (Fig. 6f). The probability of the random category tends to decrease with age (Fig. 6g).

## Discussion

In this study we compare social learning among children living in a highly linguistically and culturally diverse context with that of children in eight other societies. We refine previous research by providing statistical evidence on a range of potential learning strategies underlying children’s observable responses. We find that observable responses of ni-Vanuatu children followed the demonstrated options more often on average than those of children in other societies. Reanalyzing our Vanuatu data alongside previous data shows that neither ni-Vanuatu children, nor children from any other tested society, are more likely to follow a 3-demonstrator option over a 1-demonstrator option averaged over age. We find support for the previously found U-shaped age-pattern of *observed responses* following the 3-d option over age across societies, i.e., an increased likelihood for observed responses to follow the 3-d option over the 1-d option in comparatively young and old children, for the inferred probabilities of a majority-biased *strategy*.

ni-Vanuatu children selected a demonstrated option more frequently than children in other societies with the magnitude of this difference varying with age. This is in line with our expectations about the importance of orientation towards others^23,24^ and empirical evidence of high imitation among ni-Vanuatu children^25,27^.

We interpret the high level of following demonstrated options over an undemonstrated option across children of all ages in almost all study societies as indicative of a general reliance on social information due to its associated advantages^4,5,29^ and children’s strong inclination to learn from peers over a broad age range.

Children’s social learning likely comprises a variety of strategies, with potentially variable developmental trajectories in different communities and societies. The present study finds moderate evidence consistent with a U-shaped development of majority bias, an inverted U-shaped pattern of minority bias and random instance copying, an increase with age of copying the undemonstrated option and random demonstrator copying, and a decrease with age of a primacy bias. Which of these strategies becomes prevalent at a particular developmental timepoint might further be influenced by children’s living conditions^30^. Previous research has discussed that higher socio-economic status is associated with lower reliance on demonstrated information^13^; that adults with high-school education were more likely to endorse high conformity in children than adults with tertiary level education^25^; that household composition has no effect on social learning^31^; that less exposure to Western schooling is associated with more social information use^32–34^; that children in higher school classes conform less^35^; and that higher degrees of egalitarianism, autonomy and sharing^33^ are associated with lower reliance on demonstrated information. The interplay between the varying magnitudes of these and further conceivable learning strategies in interaction with environmental factors likely explains the ontogenetic variation in children’s reliance on demonstrated information across communities and societies.

It is important to note that some of the cross-cultural variation we report might be due to the test situation^36^ rather than to differences in social learning. While the experience of being tested on specific abstract problems and the familiarity with such situations of course varies across societies, we made every effort to develop material that used local peer demonstrators and local experimenters in Vanuatu.

Against our expectations, we find that observed responses following the 3-d option and children’s reliance on a majority-biased strategy are no higher in Vanuatu than in previously sampled societies. Whether the low levels of majority-following we report speak to the theoretical claim that a majority-bias (specifically, conformist bias) is important for the generation and maintenance of between-group differences^8^ is a topic for future work.

We found modest evidence for a gentle U-shaped pattern in age-related tendencies to follow the 3-d option, which is consistent with previous work^13^: young and old children are more likely to follow the 3-d option , while middle-aged children follow the 3-d and 1-d option at chance level. Going beyond the standard experimental measures, we provide support for a modestly U-shaped age pattern via a statistically inferred majority-biased learning strategy. Our approach allows us, for the first time, to draw a clear distinction between copying the majority with a probability of 100%, and random demonstrator copying.

We offer the following interpretation of the U-shaped pattern we found, though caution is warranted and further investigation necessary given the modesty of the U-shape and the low association of data variation with age. The found pattern might reflect a three-part developmental process in children’s information-seeking and normative motivations (see also^13^). First, young children may have an early tendency to follow the majority due to “initial spontaneous, non-rational intuitions”^13^^(p4)^. This contention is supported by studies showing a strong majority preference in 2-year-old German children^7^ as well as in 2- to 3-year old Australian children, whose strong majority preference could not be overruled by the higher efficiency of a minority model’s method^12^.

Second, around middle-childhood, children have an increased cognitive ability to represent multiple outcome scenarios simultaneously and engage in a number of different, often opposing, social learning strategies, which may decrease the signal of the majority bias. Our results show that the tendencies to follow the minority and random instance copying are at their peak in middle childhood. Also, a primacy bias is highly evident in middle childhood. Further, previous research has shown that majority-following at middle ages depends on the success^14^ and relevance of the majority’s behavior^11^, which again suggests that other biases might weaken the influence of a majority-biased strategy in middle-childhood (see also Supplementary Discussion 1.1 for further remarks).

Finally, that the majority bias resurges in older children may be driven by the developing ability to consciously use social information, based on children’s perceptions of the associated informative and normative benefits and a growing realization of the social consequences of deviating from majority norms^13^.

The development of strategies might be different if children are not naïve: Morgan et al. (2014) found that children only start at age seven to follow the majority disproportionately often, while children younger than six years follow the majority disproportionately rarely^16^.

Our manipulation checks showed that some children might not have recognized that the 3-d option was indeed shown by more demonstrators than the 1-d option (see Supplementary Discussion 1.2 for further discussion). The explanation given most often by children for their pipe choice was a pipe feature (34%), such as its position, instead of the number of demonstrators (see Supplementary Discussion 2 for all explanations). Research on children’s performance in true belief tasks shows that middle-aged children might be more likely to be confused by the pragmatics of a task^37,38^. In particular, being asked a test question that appears surprisingly easy can cause confusion^38,39^. Similarly, middle-aged children in our experimental context might have wondered about a hidden agenda behind the video demonstrations, whereas younger children followed their intuitions and older children overcame this confusion by an increased pragmatic understanding. Hence, children’s (implicit) hypotheses regarding the test situation might additionally explain the developmental pathways documented.

Besides children’s pragmatic understanding of the situation, the understanding of the demonstration video itself is likely to vary with children’s maturation, such as their attentional (e.g., Did they watch the video attentive?), numerical (e.g., Did they capture the different numbers of demonstrators?), and mnemonic (e.g., Could they remember the video content when approaching the apparatus?) abilities. The proportion of children giving a wrong answer to a manipulation check decreased from 79% in children under seven years of age to 57% in children over ten years of age. While a varying understanding of the demonstration video may have affected our results, we also consider this as an opportunity to understand how this variation influences children’s strategy use. As such, the findings on alternative learning strategies might also be the result of their differing abilities: low recall might explain the high probability of primacy bias in young children, and not understanding the manipulation might explain the high probabilities of the random category found in young children.

Future research might build on the intra-cultural data presented here by collecting socio-demographic information in addition to experimental data to not only help explain how social learning is influenced by the cultural context, but also how the *ontogeny* of social learning varies dependent on the cultural context. Specifically, within- and between-group diversity are interesting measures to consider in relation to children’s social learning because of theoretical claims about the variation-stabilizing potential of learning tendencies such as majority bias^8^.

In conclusion, our data suggest that children learn at high levels from their peers throughout their ontogeny across very different societies, with possibly even higher reliance on social information in culturally highly diverse societies. However, the age-pattern of copying social demonstrations is highly variable across societies. Majority-bias is relatively low across societies and does not coincide with variable levels of cultural diversity. Furthermore, majority bias develops in a U-shaped pattern in all tested societies, and might represent a cross-cultural universal of children’s social learning.

## Methods

### Ethics

The study protocols were approved as part of a broader project using non-invasive behavioral experiments and focusing on early child development, carried out at the Leipzig Research Center for Early Child Development in Germany, and were reviewed by the Ethics Committee of Leipzig University (reference number 169/17-ek). The committee extended the approval’s validity to Vanuatu as they had no ethical concerns about the protocol in general and considered a replication in Vanuatu as scientifically plausible. In Vanuatu, permission to carry out the research was given by the Cultural Council of the Vanuatu Cultural Center which regulates all research in the country. In line with the APA guidelines^40^, we obtained verbal assent of participating children and informed written consent from the principal of the school in which the experiments were carried out, following extensive discussion of the study protocols in all participating communities. We additionally obtained written consent from the parents of participating children for the collection of experimental data from their children (i.e., study participation, anonymous video recording, and the further usage of the data for scientific purposes such as publication of images). This study was conducted in accordance with the ethical standards laid down in the Declaration of Helsinki.

### Field site

We conducted our research on the principal island of Efate. It has a population of approximately 92,000 inhabitants^21^, belongs to the Shefa province and is also home to Vanuatu’s capital Port Vila. While there are (at least) three indigenous languages on Efate, Ifira-Mele, Nakanamanga and Nafsan^41^, many more languages indigenous to Vanuatu are spoken in a wide variety of communities today due to extensive in-migration from all over the country^42^.

Data were collected in five different schools in distinct communities. Based on the statements of the inhabitants, these communities each speak a different language. Three of them are rural communities with several hundred inhabitants, which are within 25 to 55 km of the capital and reachable in 1 to 1.5 h by public transport. The other two study locations have a few thousand inhabitants and are within 7 to 10 km of the capital, reachable in 20 to 30 min by public transport. Most of our participants live in brick houses (61%), corrugated-iron huts (22%) or houses made of local materials (15%) (data from interviews with 113 parents of participating children from ^42^). Almost all have a power supply (96%) either by solar energy (72%) or power lines (27%). Most people own smartphones (73%), some have computers (29%) or TVs (22%). Most adults participated in formal education up to primary (43%) and secondary school (50%), but not beyond. About a third of people are employed (22%) or self-employed (10%). Almost all people have gardens (89%) and use them for their subsistence (95%). The majority of ni-Vanuatu practice slash-and-burn horticulture, growing staples such as yam, taro, banana and manioc (cassava) as well as a wide variety of vegetables. This is combined with regular fishing (65%), hunting (20%), rearing livestock (54%) and purchase of market-based products. Almost half of the people we interviewed reported economic hardship within the last year (42%) (see Supplementary Methods 1 for information on all communities).

### Recruitment

Data for this experiment were collected between July and September 2018. Participants were recruited among children who had obtained prior parental consent and were attending primary school classes 1 to 4 (age range of 6 to 13 years). We prioritized children who had participated in our previous study on conformity in the same schools^35^. We collected data from about 15 children in each class with genders as balanced as possible. There were no other criteria, selection was otherwise opportunistic.

### Participants

279 children took part in the experiment. Data from 9 children had to be excluded due to experimenter error (*n* = 5), distracting/biasing environmental conditions (*n* = 2) or organizational issues (*n* = 2) (see Supplementary Methods 2.1 for a detailed report of excluded and included cases). Hence, the final sample includes data on 270 children (*age range (years)*: 5.92–13.25, *M* = 8.9, *SD* = 1.5; 140 boys; see Supplementary Methods 2.2 for the community-wise age distribution).

### Previous data

Data from a previous study by van Leeuwen and colleagues^13^ (retrieved from https://osf.io/m3ub7) is drawn from 629 children (*age range (years)*: 4–14, *M* = 8.0, *SD* = 2.5; 318 boys) from eight different societies, namely Central African Republic, Brazil, Germany, Namibia, Indonesia, Kenya, Samoa and Zambia.

### Design and study setup

We followed the study design of van Leeuwen and colleagues^13^. Instructions to the children were standardized and given in Bislama, a *lingua franca* that is understood by all participating communities and individuals. The instructions were translated from English into Bislama by a local Bislama teacher and checked for accuracy via a back-translation by another local Bislama teacher.

Two trained experimenters were present. The first experimenter was a local woman not from any of the participating communities who gave the instructions to the child. The second experimenter (also a woman) was either a white German master’s student or the first author, who took care of the video recording, counter balancing, live coding and procedure alignment.

The second experimenter called one child at a time into the study room by telling them that we would like to play a game. Nothing was revealed about the aim of the experiment. The child was welcomed by the two experimenters and asked to sit down in a place where they could see and talk to the first experimenter as well as see the laptop and box with three differently colored subsections (see Fig. 1a). The child received the instruction “I have brought a box that has three openings. If one throws a ball in the correct hole, a toy is delivered from inside the box. I will show you a movie now in which four different children will show you how to use it. Afterwards, you can use it yourself. Watch carefully”.

Then the video was shown on a laptop screen. In the video, four demonstrators approached the box one after the other (gender of models was adjusted to child’s gender). In contrast to van Leeuwen and colleagues^13^, demonstrators were from the same society as the focal child, i.e., ni-Vanuatu children (unknown to the participant). In the video, children saw two demonstrations: In the 3-demonstrator (3-d) demonstration, three children, one after another, used the same subsection A (e.g., the blue one), which releases a reward. In the 1-demonstrator (1-d) demonstration, another child uses subsection B three times (e.g., the yellow one), which releases a reward as well (see Fig. 1b). The demonstrations were counterbalanced for each community in terms of which colored subsections were used by the demonstrators and in terms of the order of 3-d and 1-d demonstrations, the 1-d demonstration always fell before or after the 3-d demonstration, never within it.

The video itself was non-verbal, however the first experimenter commented with a supporting narration according to the counterbalance condition: 3-d demonstration: “This is NAME 1, (s)he uses the blue hole. This is NAME 2, (s)he also uses the blue hole. This is NAME 3, (s)he also uses the blue hole.” and then 1-d demonstration: “This is NAME 4 (s)he uses the yellow hole. This is NAME 4 again, (s)he uses the yellow hole again. This is NAME 4 again, (s)he uses the yellow hole again.” The experimenter therefore invented four local names for the four models in the video, avoiding the child’s own name.

After the video presentation, the child received a final instruction: “Now you can use the box. Beware, you only have this one ball!” and was then given the opportunity to approach the box themselves. After children made their choice for a specific subsection, the child was asked: “Which hole did you choose? And which hole did the other children choose? And how many children chose the [*repeat colour 1 mentioned by child*] hole? And how many children chose the [*repeat colour 2 mentioned by child*] hole?” as a manipulation check.

After answering the questions, children could exchange the toy car that came out of the box for a toy car that they could take home. The child was asked not to tell their friends what happened in order to minimise the chances of them communicating with the remaining “naïve” children. After thanking them, the child went back to their class. The study was recorded with a video camera.

### Coding and reliability

The study was live coded by the second experimenter who noted the subsection choice of the child as well as any irregularities. All decisions made by the children were coded again based on the video recordings, by an intern who was blind to the hypotheses. Two cases were excluded from the reliability calculation since there was either only live or only video coding. Interrater reliability, calculated with R and the package ‘irr’^43^, was almost perfect (Cohen’s kappa: 0.978 for child’s choice). In cases of disagreement (*n* = 4), the first author made the final decision based on the video recordings.

### Statistical methods

We analyzed our data using R-Studio R version 3.6.3^44^. We implemented a runs test for detecting non-randomness to check for the possibility that children were influenced in their responses by information from already tested children: this was non-significant in all communities. Then, we analyzed data in three steps. All data and analysis scripts are available on the Open Science Framework (https://osf.io/qcs7n).

We used Bayesian statistics in our analysis and consider it for several reasons as appropriate in our study. First, Bayesian estimates are valid for any sample size. This is in contrast to Null Hypothesis Significance Testing (NHST), as can be seen from the fact that the Samoan sample had to be excluded in the previous study by van Leeuwen et al. (2018) because it was found to be highly influential in the leave-one-out cross-validation^13^. Using a Bayesian analysis allowed us to use all available data. Second, Bayesian statistics are generative models that are capable of simulating predictions without the necessity of repeated measurements. This was particularly useful in our case, as each child completed *n* = 1 trial. Still, we model different strategies. Further, we could calculate a trinomial outcome model which would, to our knowledge, not be possible in NHST.

#### Description of the ni-Vanuatu sample

To describe the observed responses of ni-Vanuatu children (*n* = 270), we followed the coding of children’s responses applied by van Leeuwen and colleagues^13^ to allow our results to be directly compared to those obtained previously.

We operationalized children’s observed responses of following one of the three experimental options as binary responses (see Fig. 1c): (1) following one of the two demonstrated options (merging 3-d and 1-d) vs. following the undemonstrated option, and (2) following the 3-d option vs. following the 1-d option (ignoring the undemonstrated option).

We examined two different subsets of our data descriptively to determine whether children’s performance on our manipulation check was associated with their observed responses. We first examined whether responses to a demonstrated versus an undemonstrated option differed between children who remembered the colors used by the demonstrators (*n* = 221) and those who did not (*n* = 49). We then examined whether children who remembered the colors used by the demonstrators *and* correctly recognized that the 3-d option was shown by more demonstrators than the 1-d option (*n* = 107) were more likely to respond to the 3-d versus the 1-d option compared to those children who did not (*n* = 163) (see Supplementary Results Sect. 1.2).

#### Comparison of the ni-Vanuatu samples to previously collected data from eight other societies

To compare ni-Vanuatu children’s observed responses with the previously obtained data from eight other societies^13^, we used a Bayesian statistical modelling approach. We modelled the binary observed responses for all children, i.e., following one of the two demonstrated options vs. following the undemonstrated option and following the 3-d vs. the 1-d option (see Fig. 1c), using a regression with a Bernoulli distribution. The model structure for following one of the two demonstrated options vs. following the undemonstrated option was:

$$L_{i } \sim Bernoulli \left( {p_{i} } \right)$$

$$logit\left( {p_{i} } \right) = \overline{a} + v_{society\_1 } + \left( {\overline{b}_{age } + v_{society\_2 } } \right)* age$$

$$v \sim multi\_normal \left( {Sigma,Rho} \right)$$

$$Sigma \sim Exponential \left( 1 \right)$$

$$Rho \sim LKJcorr \left( 2 \right)$$

$$\overline{a} \sim Normal \left( {0,1.5} \right)$$

$$\overline{b}_{age } \sim Normal \left( {0,0.5} \right)$$

$L_{i}$ indicates the 0/1 variable of a child $i$ whose observed responses followed one of the demonstrated options (vs. the undemonstrated option). The model structure for following the 3-d vs. the 1-d option was the same, except that we added a quadratic age term (analogous to the results by van Leeuwen et al.^13^):

$$logit\left( {p_{i} } \right) = \overline{a} + v_{society\_1 } + \left( {\overline{b}_{age } + v_{society\_2 } } \right)* age + \left( {\overline{b}_{{age^{2} }} + v_{society\_3 } } \right)* age^{2}$$

with

$$\overline{b}_{{age^{2} }} \sim Normal \left( {0,0.5} \right)$$

Here $L_{i}$ indicates the 0/1 variable of a child $i$ whose observed responses followed the 3-d option (vs. the 1-d option).

Sample size for the analysis on following one of the demonstrated options was *n* = 898 (269 ni-Vanuatu children excluding one child with unknown age and 629 children from the other eight societies); for the analysis on following the 3-d option it was *n* = 730 (234 ni-Vanuatu children and 496 children from the other eight societies). We estimated the posterior distribution of the models with Markov Chain Monte Carlo simulations. We sampled from the posterior distribution using the function “ulam” (R package “rethinking”^45^) and then generated model predictions based on these samples. We ran the models using Hamiltonian Monte Carlo implemented in RStan^46^. The posterior distribution is based on 4,000 samples from four chains (after 2000 adaptation steps) for a total of 8,000 samples. Importantly, our models included varying intercepts and age trends across societies to account for different age distributions of the nine societies. To calculate the strength for a difference between the distributions of the Vanuatu sample and those of the other eight societies, we calculated contrasts and the respective 89%-HPDIs.

#### Inference of learning strategies

To disentangle *observable responses* from *social learning strategies* among ni-Vanuatu children and children from eight other societies^13^ (total sample size: *n* = 898, 269 ni-Vanuatu children excluding one child with unknown age and 629 children from the other eight societies), we constructed a set of a-priori plausible strategies (see Table 1 for all strategies with explanations and expected probabilities). For each strategy, we specified the expected probability of producing each observable response. Then, we used Bayesian estimation to compute the posterior distribution of those selected strategies based on the empirically observed frequencies of the 3-d, 1-d and U-d option (see ^47^). This means that in contrast to the previous two steps, in which we operationalized children’s observed responses as binary responses and calculated binomial-outcome models, here we use a trinomial-outcome model because the unit of observation is the decision of a child with three possible outcomes: (1) 3-d option, (2) 1-d option and (3) U-d option. This requires defining a mixture likelihood over the unobserved strategies. Let *y*_*i*_ be the observed choice (1, 2 or 3) for case *i*. Then the probability of *y*_*i*_ is given by:

$$y_{i} \sim Categorical \left( {\theta_{i} } \right)$$

$$\theta_{ij} = \mathop \sum \limits_{s = 1}^{7} p_{is} \Pr {(}y_{i} = j {|}S = s)$$

$$multilogit \left( {p_{i} } \right) = \alpha_{C\left[ i \right]} + \beta_{male} male_{i} + \beta_{age1} age_{i} + \beta_{age2} age_{i}^{2}$$

where Pr(yi = j|S = s) is the strategy specific probability of choice *j* given strategy *s* (from Table 1). These probabilities are fixed, because they are aspects of the defined strategies. But the probability any child uses any particular strategy is estimated through the *p* simplex. The multilogit-linear (softmax) model for *p* allows each strategy to have its own relationship to community (through the community-specific intercept alpha), gender, and age. We use conservative priors for the component parameters in the logit-linear model:

$$\alpha_{C,j} \sim Normal\left( {a_{j} , \sigma_{j} } \right)$$

$$a \sim Normal \left( {0,1} \right)$$

$$\sigma \sim Half - Normal\left( {0,1} \right)$$

$$\beta_{male} , \beta_{age1} , \beta_{age2 } \sim Normal \left( {0,0.5} \right)$$

We again estimated the posterior distribution using Markov Chain Monte Carlo simulations with four independent chains using the R Stan version 2.19.1^46^ and the R package “rethinking”^45^. Non-centered parameterization was used for the community intercepts (alpha).

To test whether a quadratic age model is indeed superior to a linear age model, we additionally calculated a predictive model comparison using Pareto-smoothed importance sampling^28^.

## Supplementary Information

Supplementary Information.

## Data Availability

All data and analysis scripts are available on the Open Science Framework (anonymized link: https://osf.io/qcs7n).
