# Supplementary material for: Expanding the understanding of majority-bias in children’s social learning
Source: Sci Rep. 2022 Apr 25;12:6723. doi: 10.1038/s41598-022-10576-3 (PMC9038790; doi:10.1038/s41598-022-10576-3)
Supplement: Supplementary file 1 — Supplementary Information. [file 41598_2022_10576_MOESM1_ESM.docx]

SUPPLEMENTARY INFORMATION

**Expanding the Understanding of Majority-bias in Children’s Social Learning**

# Additional Acknowledgments in the Supplementary Information

We would like to thank the staff of the National Library of Vanuatu, in specific Nelly Caleb, Margaret Terry and Ernestine Kavick, for their constant support, advice and friendship.

# Results

## 1 Description of the ni-Vanuatu sample

### 1.1 Descriptive response distribution

Supplementary Table 1

*Absolute and relative frequencies of children’s observed responses for the total sample and for each community*

| Sample | 3-d | 1-d | U-d | Total |
| --- | --- | --- | --- | --- |
| All children | 110 (41%) | 124 (46%) | 36 (13%) | 270 |
| Community A | 23 (43%) | 23 (43%) | 8 (15%) | 54 |
| Community B | 21 (38%) | 29 (53%) | 5 (9%) | 55 |
| Community C | 25 (39%) | 31 (48%) | 8 (12%) | 64 |
| Community D | 13 (32%) | 15 (38%) | 12 (30%) | 40 |
| Community E | 28 (49%) | 26 (46%) | 3 (5%) | 57 |

### 1.2 Description of children’s performance on manipulation checks

1.1.2.1 Descriptive results

221 of the 270 children (82%) correctly remembered which colors were used by the demonstrators in the video. 120 of the 270 children (44% of 270) correctly remembered that the 3-d demonstrators were more children than the 1-d demonstrator. 107 (40%) of the 270 children correctly remembered both: which colors were used by the demonstrators in the video and that there were more demonstrators in the 3-d than in the 1-d demonstration (see Supplementary Figure 1).

**Supplementary Figure 1**

*Descriptive distribution of children’s performance on the manipulation checks with color recall (left), relation recall (middle) and color and relation recall together (right)*


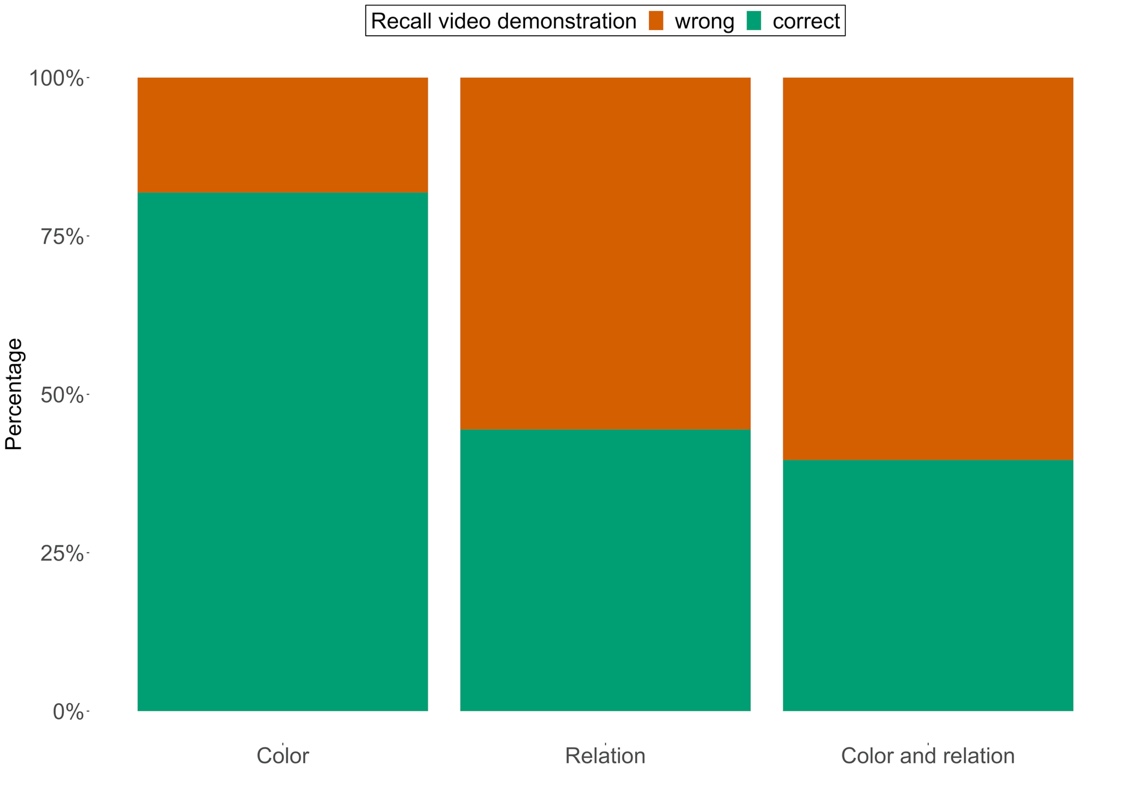


*Note. The proportion of children who answered correctly is depicted in green, the proportion of children who answered incorrectly is depicted in red.*

In the subset of children who remembered correctly which colors were used by the demonstrators in the video, 38% chose the 3-d option, 46% chose the 1-d option and 16% chose the undemonstrated option (see Supplementary Figure 2). In the subset of children who did not remember correctly which colors were used by the demonstrators in the video, 51% chose the 3-d option, 47% chose the 1-d option and 2% chose the undemonstrated option (see Supplementary Figure 2).

**Supplementary Figure 2**

*Descriptive response distribution of 3-d (blue), 1-d (yellow) and U-d option (red) for the subset of children with a correct color recall (left) and an incorrect color recall (right)*


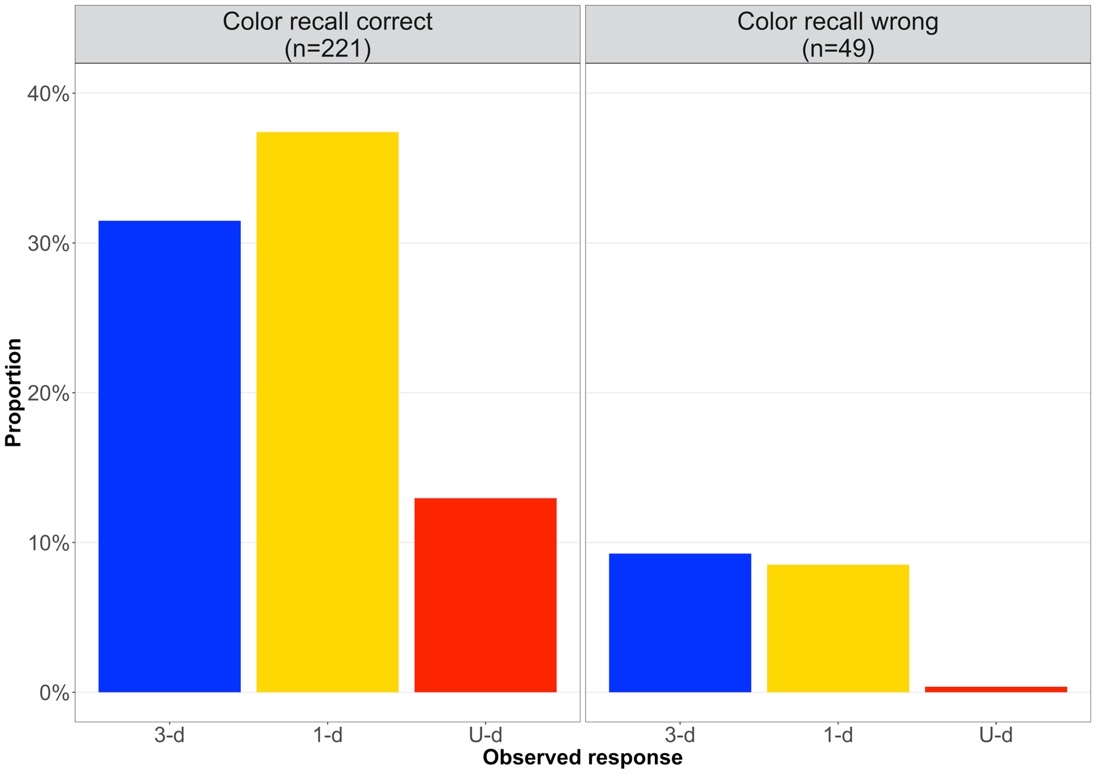


1.1.2.2 Evaluation with regard to binomial operationalization

For the (intended) use of a demonstrated option it is crucial to recognize the colors used by the demonstrators when watching the video. The distribution of observable responses of following one of the demonstrated options vs. choosing the undemonstrated option between the samples of children with a correct and incorrect color is approximately the same. The odds to choose one of the demonstrated options were 9 times larger when children did not recall the color correctly than if they recalled them correctly. We conclude that the observed responses of this subset of children with a wrong color recall show an even stronger tendency to follow one of the demonstrated options than the sample of children with a correct color recall. Our description of the overall sample could therefore, at worst, represent a too conservative picture of children’s following of a demonstrated option.

For the (intended) use of the 3-d option, it is crucial to recognize the colors used by the demonstrators (color recall) and that the 3-d demonstrators were more children than the 1-d demonstrator (relation recall) when watching the video. In the subset of children who remembered correctly which colors were used by the demonstrators and that the 3-d demonstrators were more children than the 1-d demonstrator, 38% chose the 3-d option, 48% chose the 1-d option and 14% chose the U-d option (see Supplementary Figure 3). In the subset of children who did not remember correctly which colors were used by the demonstrators in the video, 42% chose the 3-d option, 46% chose the 1-d option and 12% chose the U-d option (see Supplementary Figure 3). The distribution of observable responses of following the 3-d vs. the 1-d option between the samples of children with a correct/incorrect color and relation recall is approximately the same. If a child had a wrong color and relation recall, their odds of choosing the 3-d option were 1.2 times higher than if they had been correct. Again, we conclude that our description of the overall sample could therefore, at worst, represent a too conservative picture of children’s following of the 3-d option.

**Supplementary Figure 3**

*Descriptive response distribution of 3-d (blue), 1-d (yellow) and U-d option (red) for the subset of children with a correct color and relation recall (left) and a wrong color and relation recall (right)*


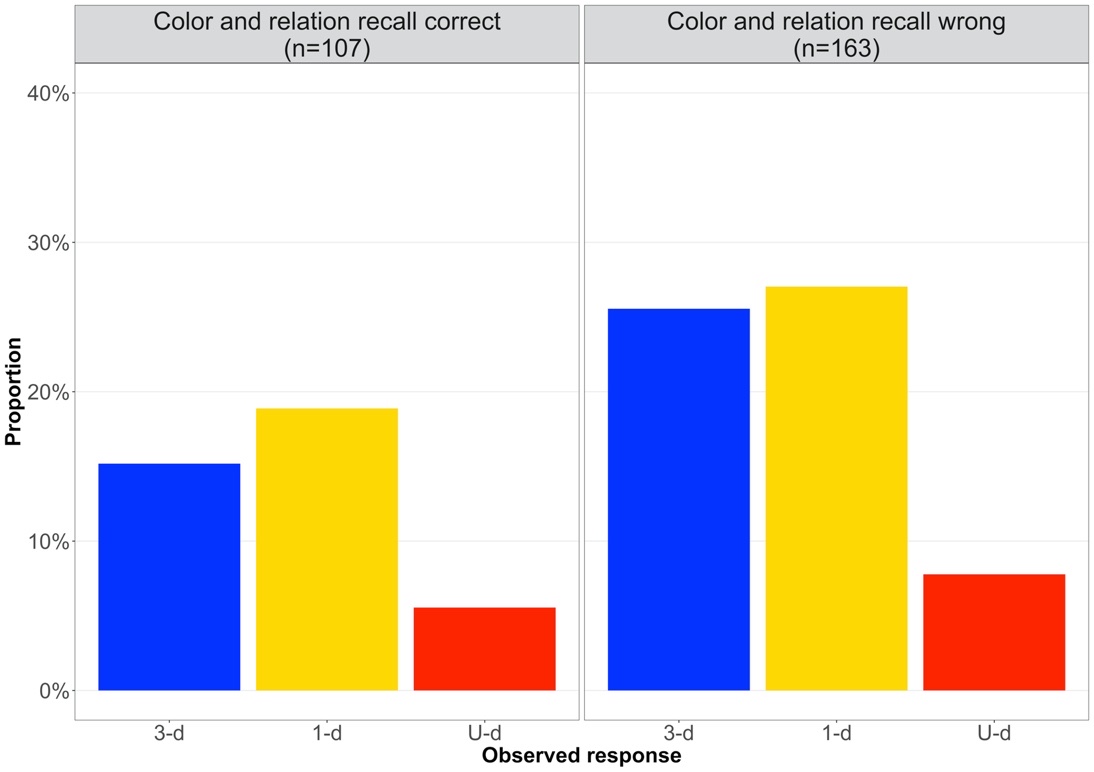


##

## 2 Comparison of the ni-Vanuatu sample with previously published data:

### 2.1 Descriptive response distribution

Supplementary Figure 4

*Descriptive response distribution of 3-d (blue), 1-d (yellow) and an U-d option (red) for the eight societies that have been sampled by van Leeuwen and colleagues*^1^


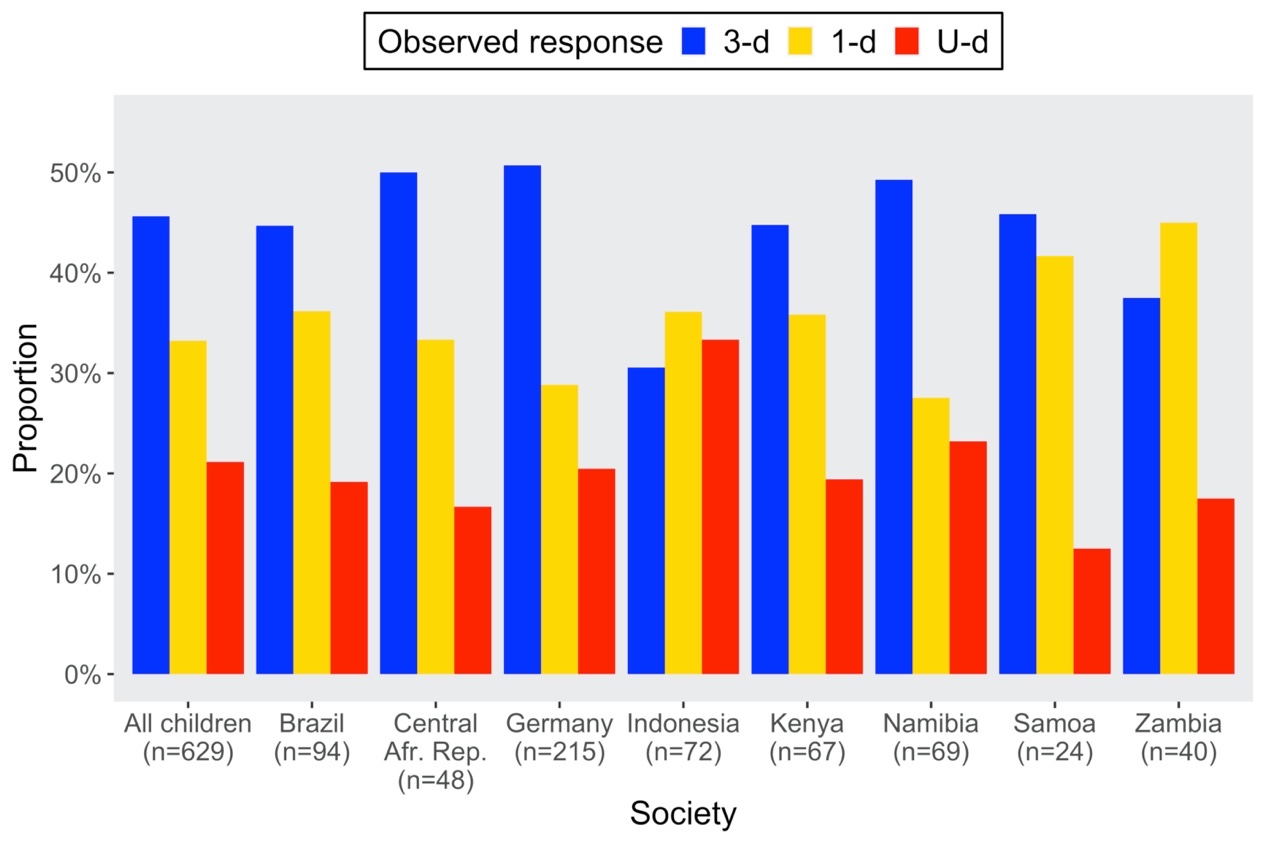


### 2.2 Model coefficients

**Supplementary Table 2**

*Coefficients of the model on using one of the demonstrated options vs. the undemonstrated option with mean, standard deviation and the borders of the 89% percentile interval*

|  | Mean | SD | PI (5.5) | PI (94.5) |
| --- | --- | --- | --- | --- |
| a_bar | 1.46 | 0.18 | 1.17 | 1.75 |
| bA_bar | -0.06 | 0.15 | -0.29 | 0.19 |
| Sigma[1] | 0.41 | 0.19 | 0.15 | 0.73 |
| Sigma[2] | 0.31 | 0.17 | 0.07 | 0.6 |
| L_Rho[1,1] | 1 | 0 | 1 | 1 |
| L_Rho[1,2] | 0 | 0 | 0 | 0 |
| L_Rho[2,1] | 0.3 | 0.39 | -0.4 | 0.84 |
| L_Rho[2,2] | 0.85 | 0.16 | 0.54 | 1 |
| v[Samoa,1] | 0.17 | 0.39 | -0.38 | 0.81 |
| v[Samoa,2] | 0.03 | 0.3 | -0.43 | 0.52 |
| v[Brazil,1] | 0.14 | 0.3 | -0.29 | 0.63 |
| v[Brazil,2] | 0.26 | 0.26 | -0.07 | 0.72 |
| v[Indonesia,1] | -0.62 | 0.36 | -1.22 | -0.08 |
| v[Indonesia,2] | -0.3 | 0.32 | -0.88 | 0.12 |
| v[CAR,1] | 0.13 | 0.31 | -0.32 | 0.66 |
| v[CAR,2] | 0.13 | 0.28 | -0.25 | 0.63 |
| v[Kenya,1] | 0.02 | 0.27 | -0.41 | 0.45 |
| v[Kenya,2] | 0.07 | 0.26 | -0.3 | 0.5 |
| v[Zambia,1] | 0.06 | 0.3 | -0.4 | 0.55 |
| v[Zambia,2] | 0.01 | 0.24 | -0.38 | 0.39 |
| v[Germany,1] | 0 | 0.23 | -0.35 | 0.37 |
| v[Germany,2] | -0.24 | 0.19 | -0.57 | 0.02 |
| v[Vanuatu,1] | 0.34 | 0.24 | 0 | 0.74 |
| v[Vanuatu,2] | 0.09 | 0.21 | -0.22 | 0.44 |
| v[Namibia,1] | -0.11 | 0.26 | -0.53 | 0.3 |
| v[Namibia,2] | -0.03 | 0.2 | -0.35 | 0.28 |

**Supplementary Table 3**

*Coefficients of the model on using the 3-d vs. the 1-d option with mean, standard deviation and the borders of the 89% percentile interval*

|  | Mean | SD | PI (5.5) | PI (94.5) |
| --- | --- | --- | --- | --- |
| a_bar | 0.05 | 0.14 | -0.16 | 0.27 |
| bA_bar | 0.03 | 0.12 | -0.14 | 0.22 |
| bA2_bar | 0.15 | 0.1 | 0 | 0.3 |
| Sigma[1] | 0.19 | 0.13 | 0.02 | 0.43 |
| Sigma[2] | 0.17 | 0.15 | 0.01 | 0.44 |
| Sigma[3] | 0.12 | 0.11 | 0.01 | 0.31 |
| L_Rho[1,1] | 1 | 0 | 1 | 1 |
| L_Rho[1,2] | 0 | 0 | 0 | 0 |
| L_Rho[1,3] | 0 | 0 | 0 | 0 |
| L_Rho[2,1] | -0.01 | 0.4 | -0.66 | 0.64 |
| L_Rho[2,2] | 0.91 | 0.11 | 0.67 | 1 |
| L_Rho[2,3] | 0 | 0 | 0 | 0 |
| L_Rho[3,1] | -0.01 | 0.41 | -0.66 | 0.65 |
| L_Rho[3,2] | -0.02 | 0.41 | -0.67 | 0.65 |
| L_Rho[3,3] | 0.8 | 0.16 | 0.48 | 0.99 |
| v[Samoa,1] | -0.02 | 0.2 | -0.34 | 0.28 |
| v[Samoa,2] | -0.01 | 0.18 | -0.28 | 0.25 |
| v[Samoa,3] | 0 | 0.13 | -0.2 | 0.19 |
| v[Brazil,1] | 0 | 0.16 | -0.25 | 0.27 |
| v[Brazil,2] | 0.03 | 0.15 | -0.18 | 0.28 |
| v[Brazil,3] | 0 | 0.11 | -0.16 | 0.17 |
| v[Indonesia,1] | -0.07 | 0.19 | -0.41 | 0.18 |
| v[Indonesia,2] | 0.09 | 0.19 | -0.13 | 0.44 |
| v[Indonesia,3] | -0.05 | 0.14 | -0.31 | 0.1 |
| v[CAR,1] | 0.05 | 0.19 | -0.21 | 0.38 |
| v[CAR,2] | -0.1 | 0.21 | -0.49 | 0.13 |
| v[CAR,3] | 0.07 | 0.17 | -0.1 | 0.37 |
| v[Kenya,1] | 0.04 | 0.17 | -0.21 | 0.33 |
| **Supplementary Table 3 continued** | | | | |
|  | Mean | SD | PI (5.5) | PI (94.5) |
| v[Kenya,2] | 0.08 | 0.19 | -0.14 | 0.45 |
| v[Kenya,3] | -0.02 | 0.14 | -0.25 | 0.15 |
| v[Zambia,1] | -0.09 | 0.2 | -0.46 | 0.16 |
| v[Zambia,2] | 0.06 | 0.18 | -0.15 | 0.4 |
| v[Zambia,3] | 0.01 | 0.12 | -0.16 | 0.2 |
| v[Germany,1] | 0.13 | 0.17 | -0.07 | 0.44 |
| v[Germany,2] | -0.04 | 0.13 | -0.26 | 0.13 |
| v[Germany,3] | 0.01 | 0.09 | -0.12 | 0.17 |
| v[Vanuatu,1] | -0.12 | 0.16 | -0.4 | 0.07 |
| v[Vanuatu,2] | -0.05 | 0.15 | -0.33 | 0.14 |
| v[Vanuatu,3] | -0.05 | 0.12 | -0.28 | 0.09 |
| v[Namibia,1] | 0.07 | 0.18 | -0.18 | 0.39 |
| v[Namibia,2] | -0.05 | 0.15 | -0.32 | 0.15 |
| v[Namibia,3] | 0.05 | 0.11 | -0.09 | 0.25 |

### 2.3 Separate analysis of the Vanuatu sample

**Supplementary Table 4**

*Coefficients of the model on ni-Vanuatu children alone using the 3-d vs. the 1-d option with mean, standard deviation and the borders of the 89% percentile interval*

|  | Mean | SD | PI (5.5) | PI (94.5) |
| --- | --- | --- | --- | --- |
| a_bar | -0.09 | 0.68 | -1.1 | 0.98 |
| bA_bar | 0.01 | 0.32 | -0.49 | 0.51 |
| bA2_bar | 0 | 0.33 | -0.53 | 0.54 |
| Sigma[1] | 0.79 | 0.78 | 0.04 | 2.28 |
| Sigma[2] | 0.58 | 0.64 | 0.03 | 1.78 |
| Sigma[3] | 0.57 | 0.61 | 0.03 | 1.78 |
| L_Rho[1,1] | 1 | 0 | 1 | 1 |
| L_Rho[1,2] | 0 | 0 | 0 | 0 |
| L_Rho[1,3] | 0 | 0 | 0 | 0 |
| L_Rho[2,1] | 0 | 0.42 | -0.66 | 0.67 |
| L_Rho[2,2] | 0.9 | 0.12 | 0.65 | 1 |
| L_Rho[2,3] | 0 | 0 | 0 | 0 |
| L_Rho[3,1] | 0 | 0.4 | -0.64 | 0.64 |
| L_Rho[3,2] | 0 | 0.41 | -0.66 | 0.66 |
| L_Rho[3,3] | 0.8 | 0.16 | 0.5 | 0.99 |
| v[1,1] | -0.04 | 0.67 | -1.12 | 0.96 |
| v[1,2] | 0 | 0.31 | -0.51 | 0.51 |
| v[1,3] | 0 | 0.33 | -0.53 | 0.54 |

**Supplementary Figure 5**

*The figure shows the age-trajectory of ni-Vanuatu children’s reliance on the 3-d vs. the 1-d option as predicted by the Bayesian multilevel model based on the ni-Vanuatu data alone. The horizontal dotted line represents the expected proportion if children’s observed responses were random (probability of 0.5). The light gray lines are 500 lines sampled from the posterior distribution showing the uncertainty of the predicted trajectory.*


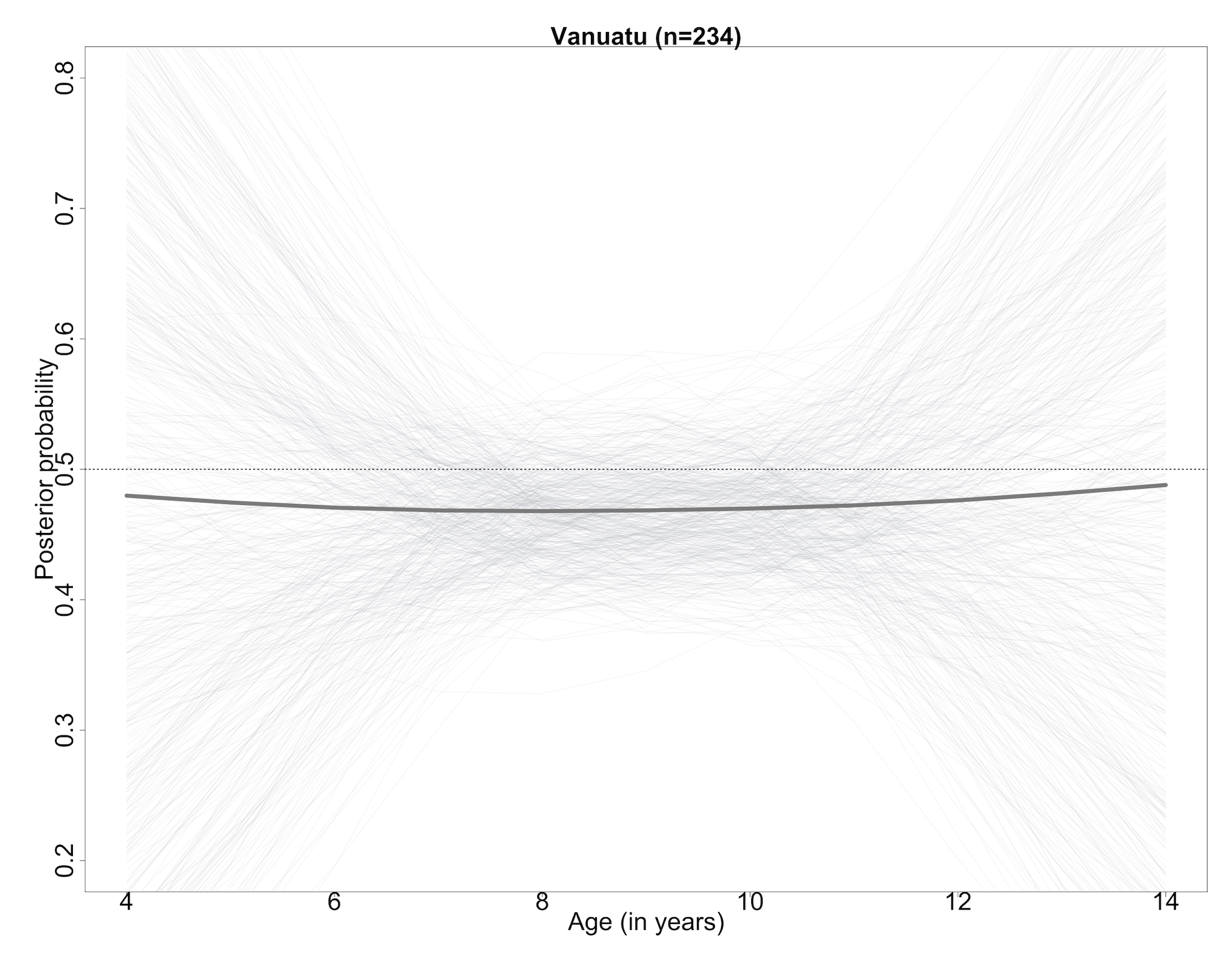


### 2.4 Quadratic age term: Sum of the global average and the society specific effects

**Supplementary Table 5**

*89% Highest posterior density interval of the sum of the varying effect offset of the quadratic age term of the global average (*$\bar{b}_{{age}^{2}}$*) and society specific effects (*$v_{society}$*)*

| **Sample** | **89% HPDI [5.5%, 94.5%]** |
| --- | --- |
| Samoa | [-0.06; 0.39] |
| Brazil | [-0.01; 0.35] |
| Indonesia | [-0.13; 0.34] |
| CAR | [-0.04; 0.46] |
| Kenya | [-0.10; 0.38] |
| Zambia | [-0.04; 0.36] |
| Germany | [0.03; 0.31] |
| Vanuatu | [-0.10; 0.32] |
| Namibia | [0.00; 0.38] |

### 2.5 Age-specific contrasts

**Supplementary Figure 6**

*Posterior density distributions of the Bayesian multilevel models for observed responses following one of the demonstrated options vs. the undemonstrated option for each society where each is a deviation from the Vanuatu data at a specific age.*


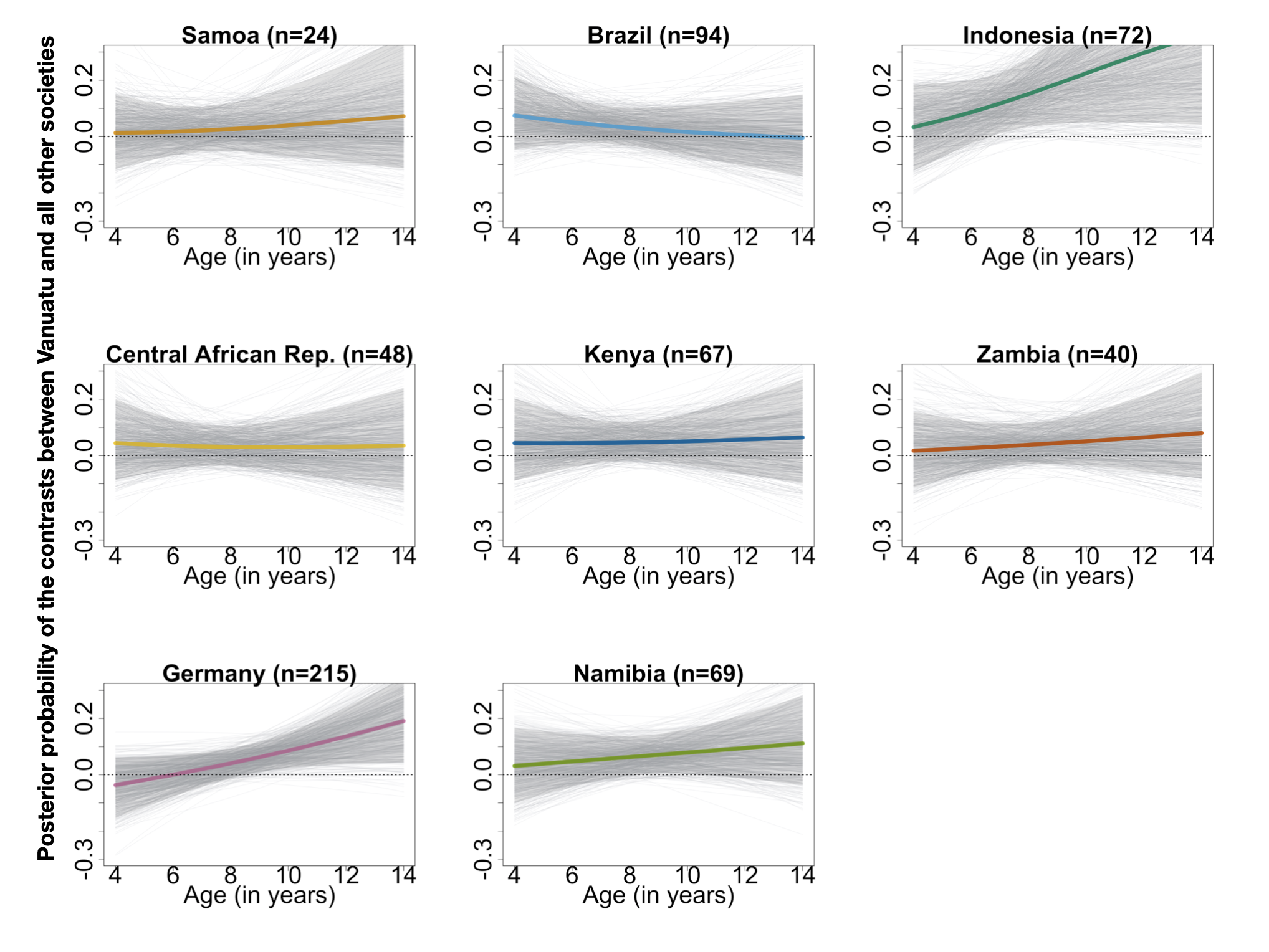


*Note. The horizontal dotted line represents no deviation between Vanuatu and a society. The light gray lines are 500 lines sampled from the posterior distribution showing the uncertainty of the predicted trajectories. Shaded areas represent 89% posterior compatibility intervals.*

**Supplementary Figure 7**

*Posterior density distributions of the Bayesian multilevel models for observed responses for following the 3-d option vs. the 1-d option for each society where each is a deviation from the Vanuatu data at a specific age.*


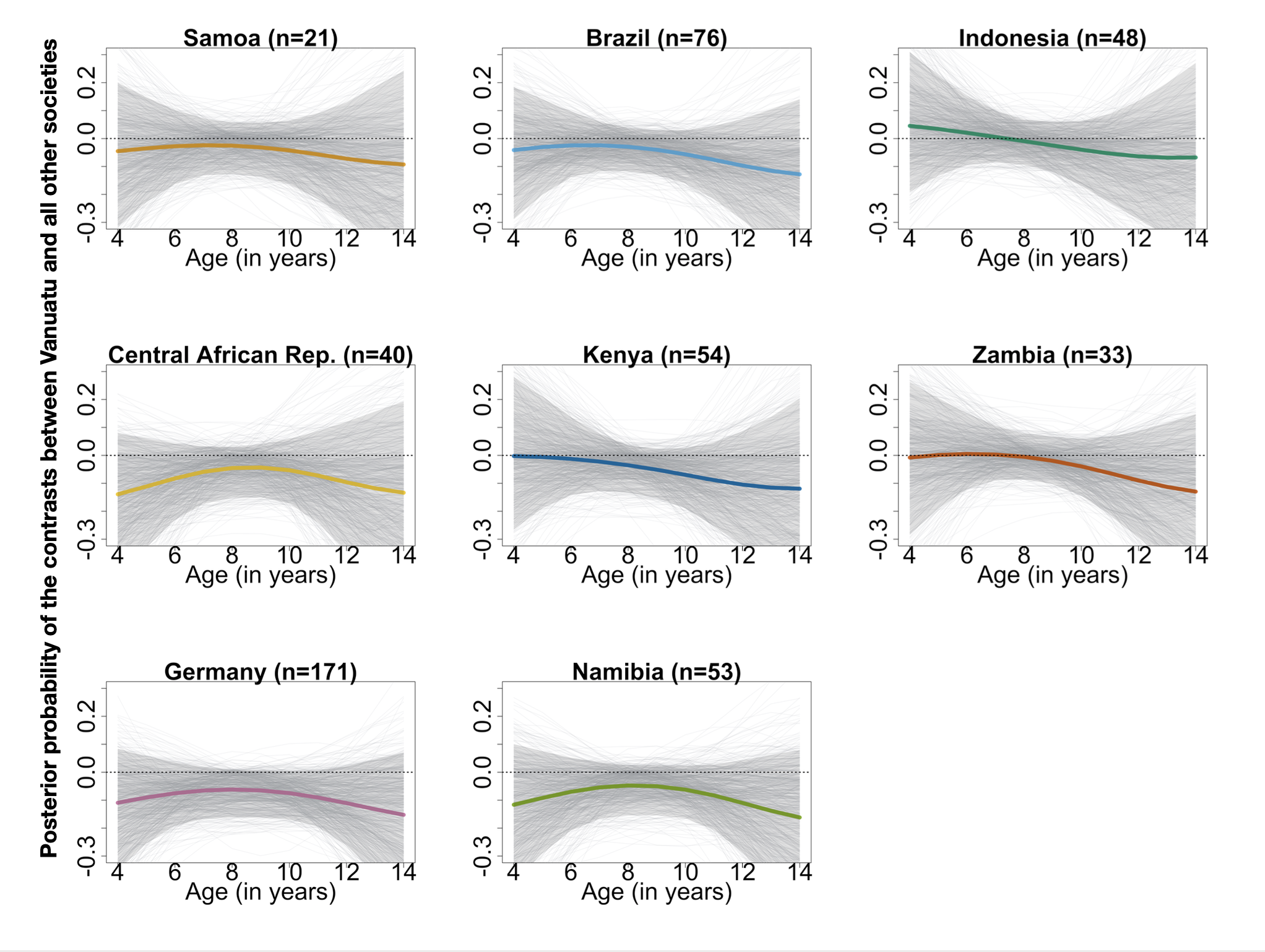


*Note. The horizontal dotted line represents no deviation between Vanuatu and a society. The light gray lines are 500 lines sampled from the posterior distribution showing the uncertainty of the predicted trajectories. Shaded areas represent 89% posterior compatibility intervals.*

## 3 Learning strategies

### 3.1 Posterior means and percentile intervals of each strategy by society

**Supplementary Table 6**

*Posterior means and borders of the 89% Percentile Intervals of each strategy by society*

| Society | Strategy | | | | | | |
| --- | --- | --- | --- | --- | --- | --- | --- |
|  | Majority bias | Minority bias | Undemons-trated | Primacy bias | Random demonstrator | Random instance | Random |
| Brazil | 0.11  [0.00;0.37] | 0.05  [0.00;0.15] | 0.13  [0.01;0.28] | 0.31  [0.08;0.53] | 0.11  [0.00;0.42] | 0.08  [0.00;0.27] | 0.21  [0.00;0.61] |
| CAR | 0.18  [0.00;0.57] | 0.07  [0.00;0.22] | 0.13  [0.01;0.28] | 0.14  [0.01;0.32] | 0.16  [0.00;0.59] | 0.12  [0.00;0.40] | 0.20  [0.00;0.65] |
| Germany | 0.13  [0.01;0.39] | 0.03  [0.00;0.10] | 0.17  [0.03;0.32] | 0.37  [0.13;0.60] | 0.12  [0.00;0.40] | 0.07  [0.00;0.22] | 0.11  [0.00;0.38] |
| Indonesia | 0.09  [0.00;0.30] | 0.06  [0.00;0.19] | 0.22  [0.01;0.52] | 0.14  [0.01;0.33] | 0.09  [0.00;0.35] | 0.08  [0.00;0.28] | 0.33  [0.01;0.87] |
| Kenya | 0.14  [0.00;0.47] | 0.07  [0.00;0.20] | 0.13  [0.01;0.30] | 0.10  [0.01;0.25] | 0.15  [0.00;0.55] | 0.12  [0.00;0.44] | 0.28  [0.01;0.78] |
| Namibia | 0.17  [0.00;0.57] | 0.06  [0.00;0.20] | 0.15  [0.02;0.31] | 0.10  [0.01;0.24] | 0.16  [0.00;0.61] | 0.10  [0.00;0.36] | 0.25  [0.01;0.72] |
| Samoa | 0.13  [0.00;0.45] | 0.08  [0.00;0.24] | 0.12  [0.01;0.29] | 0.24  [0.03;0.51] | 0.14  [0.00;0.52] | 0.11  [0.00;0.40] | 0.18  [0.00;0.57] |
| Vanuatu | 0.09  [0.00;0.31] | 0.07  [0.00;0.20] | 0.09  [0.01;0.20] | 0.38  [0.12;0.57] | 0.09  [0.00;0.37] | 0.09  [0.00;0.28] | 0.18  [0.00;0.56] |
| Zambia | 0.13  [0.00;0.46] | 0.10  [0.00;0.31] | 0.13  [0.01;0.29] | 0.16  [0.02;0.36] | 0.13  [0.00;0.51] | 0.12  [0.00;0.40] | 0.23  [0.01;0.70] |

### 3.2 Alternative illustrations

**Supplementary Figure 8**

*Inferred* *probabilities of all seven strategies, i.e., following the majority (blue), minority (yellow), choosing the undemonstrated option (red), following the first shown option (beige), using the unbiased strategy (dark green), using the random instance strategy (light green) and the random category (grey) over age based on posterior distributions from a Bayesian trinomial-outcome model*


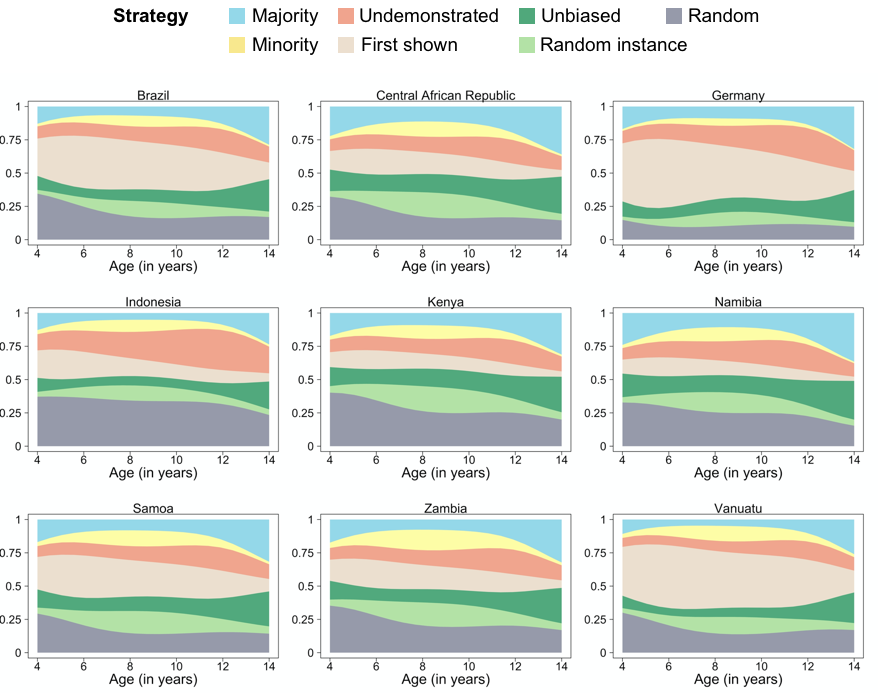


**Supplementary Figure 9**

*Inferred* *probabilities of the three strategies of primary interest, i.e., following the majority (blue), minority (yellow) and choosing the undemonstrated option (red) over age based on posterior distributions from a Bayesian trinomial-outcome model, depicted are 3 out of the 7 considered strategies*


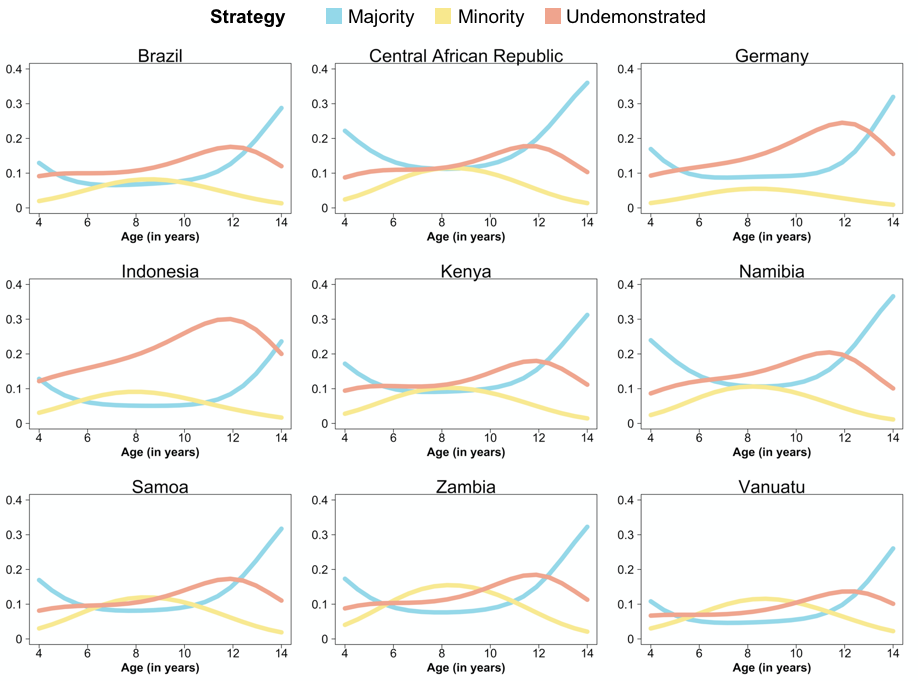


**Supplementary Figure 10**

*Inferred* *probabilities of the four other strategies, i.e., following the first shown option (beige), using the unbiased strategy (dark green), using the random instance strategy (light green) and the random category (grey) over age based on posterior distributions from a Bayesian trinomial-outcome model, depicted are 4 out of the 7 considered strategies*


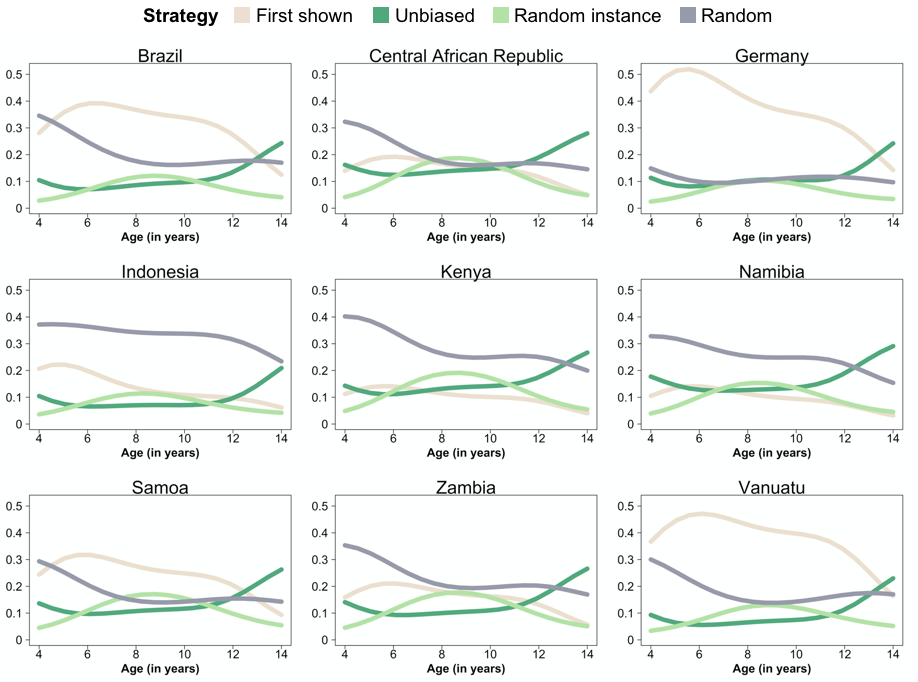


### 3.3 Uncertainty of the U-shaped pattern of the majority bias strategy

**Supplementary Figure 11**

*Inferred probabilities of following the majority and their 89% compatibility regions over age based on posterior distributions from a Bayesian trinomial-outcome model*


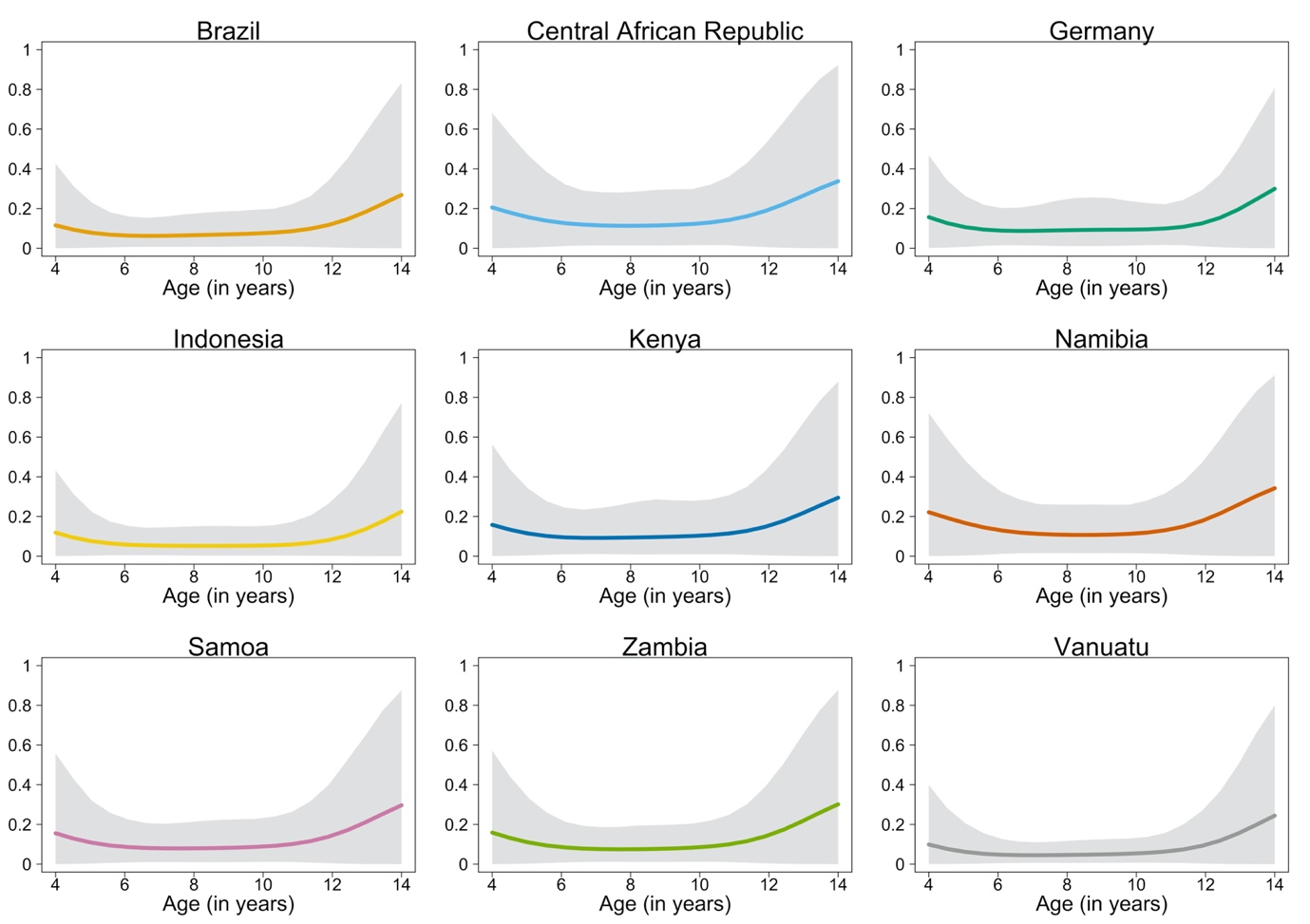


# Discussion

## 1 Choice of 3-d option

### 1.1 More on the U-shape

Around middle-childhood, children have an increased ability to engage in a number of different often opposing social learning heuristics which decreases the signal of the majority bias. Our results of plausible underlying learning strategies show that the tendencies to (a) follow the minority and (b) copy based on the number of instances are at their peak in middle childhood. Further, although decreasing over age, a primacy bias (c) is in most of the investigated societies still at high rates in middle childhood (Brazil, Germany, Indonesia, Zambia, Samoa, Vanuatu). We will discuss the role of these biases in the following.

Children might copy the behavior of the minority demonstrator (a) as they display consistency over time^3,4^, which comes along with the attribution of certainty, competence, and commitment (for a review see^4^). Additionally, the participating child might feel affiliated to the minority demonstrator as they both appear to stand alone in relation to the remaining three protagonists constituting the majority, and are hence in a comparable situation^3,5^. Both, source consistency and source similarity might lead to an informative motivation of children. This is supported by the fact that 50% of the children who children stated that they “wanted to follow other children” had previously selected the 1-d option.

Selecting the 1-d and 3-d option equally often, can also be rooted in copying according to the number of demonstrations (b). While previous studies have shown an influence of the number of demonstrations especially in young children of 2- to 3 years^6,7^, our results indicate that this learning heuristic might still be applied by children in middle childhood.

Lastly, a strong primacy bias (c) can explain the pattern of almost equal numbers of choices for the 3-d and 1-d option as the order of the 1-d and 3-d demonstration was counterbalanced across children. A primacy bias means that once an opinion is formed, the following information is perceived as less important. The odds for ni-Vanuatu children to follow one of the two options were 9.4 times larger when it was demonstrated first than when it was demonstrated second (see also^1^ for similar effects).

### 1.2 Manipulation check results

Only 44% of the ni-Vanuatu children correctly remembered that the 3-d children were more than the 1-d children (due to missing manipulation checks in the previous sample, we cannot make statements about the other eight societies). Hence, though most children remembered correctly which pipes were used by the demonstrators (82%), the different numbers of demonstrators might not have been salient enough to some children. This is supported by the fact that the explanation given most often by children for their pipe choice is a pipe feature (34%, see Supplementary Table 7). The low saliency of the manipulation might be explained by the artificial setting of the experiment^8^ and ni-Vanuatu children’s little experience of being questioned on specific abstract problems^9^.

We tried to mitigate these effects by having a local experimenter and ni-Vanuatu children as demonstrators. Further, ni-Vanuatu children study in a Western school system which is marked by intensive instruction^10^, and might already have been familiarized with instructions and explanations as we used them. Hence, it might be, that instead of a low saliency of the manipulation, just a low recall explains the poor performance in manipulation checks. In this case, children may not have remembered the manipulation but were still influenced by it.

## 2 Explanations of children and parents for their pipe choice

### 2.1 Explanations given by children

To get an idea of children’s intentions for selecting a certain pipe, we asked children (*n* = 68) from 2 communities in a non-representative survey for the reasons of their pipe choice.

**Supplementary Table 7**

*Absolute and relative frequencies of explanations given by ni-Vanuatu children depending on their pipe choice (n=68)*

| **Explanation** | **Example** | **3-d option chosen** | **1-d option chosen** | **U-d option chosen** | **total** |
| --- | --- | --- | --- | --- | --- |
| Pipe feature | “The pipe is in the middle.”  “The pipe is straight.”  “It is my color in the school team.”  “It is the color of the sky.” | 3 (13%) | 16 (70%) | 4 (17%) | 23 (34%) |
| Follow other children | “I followed one of the other children.”  “I followed the other children.” | 8 (50%) | 8 (50%) | 0 | 16 (24%) |
| Pipe has the truck | “I like the truck.”  “Because the truck is inside.”  “I wanted to get a truck.” | 5 (56%) | 3 (33%) | 1 (11%) | 9 (13%) |
| Pipe was not used before | “Because it was never used.” | 0 | 0 | 6 (100%) | 6 (9%) |
| Because of the movie | “I saw that the truck is nice.” | 1 (25%) | 2 (50%) | 1 (25%) | 4 (6%) |
| The child is just one | “She is just one.” | 0 | 0 | 1 (100%) | 1 (1%) |
| Unknown | “I just chose it” | 4 (44%) | 3 (33%) | 2 (22%) | 9 (13%) |

### 2.2 Explanations given by parents of participating children

We asked parents of participating children, why they would use a certain pipe themselves (*n* = 85) and why their child should use a certain pipe (*n* = 65) (data from interviews with 113 parents of participating children from [blinded for review], unpublished PhD).

The main reasons for parent’s choices of a certain pipe were that the pipe had a specific feature (*n* = 38) or that it was not used yet (*n* = 12). 36.5% (31 of 85) of the parents justify their own choice by the use social information (with reasons like “because of the movie” or “used by many”), 51.6% (*n* = 16) of them in a way to then use the undemonstrated pipe (“not used yet”).

Concerning their child’s choices, pipe features (*n* = 26) and the child’s independence (*n* = 15) are most often mentioned. 21.5% (14 of 65) of the parents, who gave an answer on which pipe their child should use, suggest to use social information, 35.7% (*n* = 5) of them in a way to then use the undemonstrated pipe.

# Methods

## 1 Socio-economic data from communities

**Supplementary Table 8**

*Results from interviews with parents of participating children. All numbers are relative frequencies in % if not otherwise specified.*

| Community | A  (n=18) | B  (n=17) | C  (n=29) | D  (n=19) | E  (n=30) | total  (n=113) |
| --- | --- | --- | --- | --- | --- | --- |
| House Kind |  | | | | | |
| Bricks | 33 | 41 | 86 | 32 | 86 | 61 |
| Kappa | 17 | 18 | 11 | 58 | 14 | 22 |
| Local house | 50 | 41 | 0 | 0 | 0 | 15 |
| Electricity available | 100 | 88 | 97 | 100 | 97 | 96 |
| Kind of electricity | | | | | | |
| Power line | 0 | 92 | 75 | 0 | 3 | 27 |
| Solar | 100 | 8 | 25 | 100 | 96 | 72 |
| Owns smartphone | 67 | 82 | 66 | 89 | 69 | 73 |
| Owns computer | 39 | 18 | 41 | 42 | 10 | 29 |
| Owns TV | 0 | 24 | 48 | 21 | 10 | 22 |
| Made holiday in a foreign country | 6 | 0 | 10 | 11 | 7 | 7 |
| Highest level of education | | | | | | |
| primary school | 39 | 29 | 46 | 53 | 45 | 43 |
| secondary school | 61 | 71 | 43 | 42 | 41 | 50 |
| college | 0 | 0 | 0 | 5 | 3 | 2 |
| university | 0 | 0 | 11 | 0 | 10 | 6 |
| Works out of home | 28 | 35 | 52 | 37 | 10 | 32 |

**Supplementary Table 8 continued**

| Community | A  (n=18) | B  (n=17) | C  (n=29) | D  (n=19) | E  (n=30) | total  (n=113) |
| --- | --- | --- | --- | --- | --- | --- |
| Kind of work | | | | | | |
| employed | 22 | 18 | 38 | 26 | 7 | 22 |
| autonomous | 6 | 18 | 14 | 11 | 3 | 10 |
| at home | 72 | 47 | 48 | 58 | 83 | 62 |
| no work | 0 | 18 | 0 | 5 | 7 | 5 |
| Employed time |  |  |  |  |  |  |
| fulltime | 22 | 29 | 41 | 21 | 7 | 24 |
| seasonally | 6 | 0 | 4 | 0 | 0 | 2 |
| Uses garden for subsistence | 100 | 94 | 92 | 89 | 97 | 95 |
| Fishes for subsistence | 67 | 65 | 45 | 89 | 69 | 65 |
| Hunts for subsistence | 17 | 18 | 21 | 11 | 28 | 20 |
| Has livestock for subsistence | 72 | 29 | 66 | 16 | 69 | 54 |
| Experienced hardship | 67 | 88 | 69 | 84 | 52 | 70 |
| Experienced hardship within the last year | 44 | 50 | 32 | 58 | 34 | 42 |
| Owns garden | 100 | 88 | 86 | 95 | 83 | 89 |

## 2 Participants

### 2.1 Detailed report of excluded and included cases

**Supplementary Table 9**

*In- and excluded cases*

| **Decision** | **Topic** | **Issue** | **n** |
| --- | --- | --- | --- |
| Inclusion | Video Demonstration | Wrong color mentioned through experimenter while showing the video but corrects herself immediately | 1 |
|  |  | Change between boys/girls video immediately after video started | 2 |
|  |  | One majority child named after friend of subject | 1 |
|  |  | Experimenter points on laptop during video demonstration | 2 |
|  |  | Interruption by another person after showing the video | 1 |
|  | Response | Subject looks at experimenter for help when entering the ball | NA |
|  |  | Subject very hesitant, repeatedly looking at the experimenter for help, throws ball after very long time | NA |
|  |  | Subject looks at experimenter for help, experimenter explains that he/she should do it like the children in the movie | 1 |
|  | Environment | A lot of noise from outside | 63 |
|  |  | School bell rings during videos | 1 |
|  |  | Loud singing in the neighboring classroom, therefore short break before video demonstration | 1 |
|  |  | Experimenter plays around with yellow folder during study | 1 |
|  |  | Headmistress watches | 1 |
|  | Box/Technical Problems | Toy does not come out when subject has entered the ball | 7 |
|  |  | Toy was put into the box when subject was present in the room | 1 |
|  |  | You cannot see in which hole the subject threw the ball on video | 1 |
|  |  | Not recorded | 1 |
|  |  | Recorded after introduction | 2 |
|  |  | One box wall fell down | 1 |

| **Supplementary Table 9 continued** | | | |
| --- | --- | --- | --- |
| **Decision** | **Topic** | **Issue** | **n** |
| Inclusion | Miscellaneous | Experimenter bobs | 1 |
|  |  | Child said purple instead of blue | 5 |
|  |  | Experimenter covers toy cars with cardboard to focus attention of the child | 1 |
|  |  | Experimenter wanted to show video too early | 1 |
|  |  | Experimenter bobs | 1 |
| Exclusion | Miscellaneous | Video of the opposite gender shown | 1 |
|  |  | Minority child was named differently during the 3 demonstrations | 1 |
|  |  | One majority child named after subject | 1 |
|  |  | Subject was sitting on the wrong place and probably wanted to throw the ball in yellow, after moving she decided to blue | 1 |
|  |  | E1 says something like yellow shortly before response | 1 |
|  |  | Someone is talking about colors in the background right before the response of the child | 1 |
|  |  | Same child was invited twice, just first response was taken | 2 |
|  |  | Video shown at home, a lot of people around, first videos were shown without saying names | 1 |

### 2.2 Community-wise participant description

**Supplementary Table 10**

*Sample composition: age distribution of children by community for the total sample*

| Community | N (male) | mean age | sd age | range age |
| --- | --- | --- | --- | --- |
| A | 54 (28) | 8.7 | 1.66 | 6.08,13.25 |
| B | 55 (27) | 9.2 | 1.4 | 5.92,12.00 |
| C | 64 (34) | 9.1 | 1.41 | 6.33,12.25 |
| D | 40 (22) | 8.6 | 1.55 | 6.00,12.00 |
| E | 57 (29) | 8.9 | 1.69 | 5.92,12.50 |

**Supplementary Figure 12**

*Age of sampled children by community*
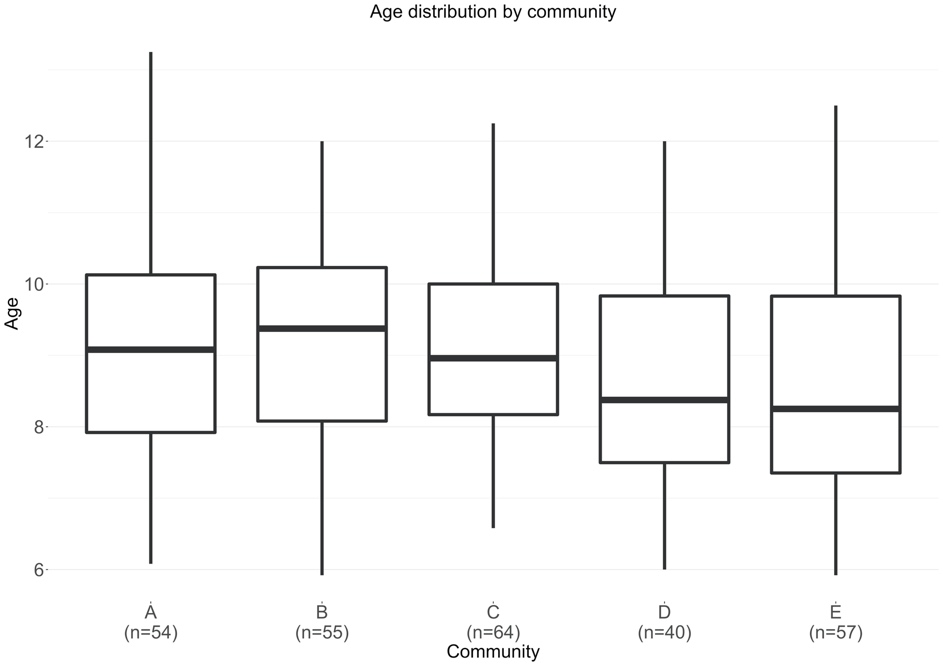


*Note. The boxes represent the interquartile range (IQR), the bold, horizontal lines within the boxes are medians, the upper vertical lines attached to the boxes extend from the hinge to the largest value no further than 1.5 * IQR from the hinge, the lower vertical lines attached to the boxes extend from the hinge to the smallest value at most 1.5 * IQR of the hinge.*
